# An interdisciplinary approach to characterize peanut‐allergic patients—First data from the FOOD@ consortium

**DOI:** 10.1002/clt2.12197

**Published:** 2022-10-05

**Authors:** Margitta Worm, Aikaterina Alexiou, Veronika Höfer, Till Birkner, Alexander C. S. N. Jeanrenaud, Florent Fauchère, Kristijan Pazur, Carolin Steinert, Aleix Arnau‐Soler, Priyanka Banerjee, Andreas Diefenbach, Josefine Dobbertin‐Welsch, Sabine Dölle‐Bierke, Wojciech Francuzik, Ahla Ghauri, Stephanie Heller, Birgit Kalb, Ulrike Löber, Ingo Marenholz, Lajos Markó, Jörg Scheffel, Olena Potapenko, Stephanie Roll, Susanne Lau, Young‐Ae Lee, Julian Braun, Andreas Thiel, Magda Babina, Sabine Altrichter, Sofia Kirke Forslund, Kirsten Beyer

**Affiliations:** ^1^ Division of Allergy and Immunology Department of Dermatology, Venerology and Allergy Charité – Universitätsmedizin Berlin Freie Universität Berlin and Humboldt‐Universität zu Berlin Berlin Germany; ^2^ Experimental and Clinical Research Center A Cooperation of Charité‐Universitätsmedizin Berlin Max Delbrück Center for Molecular Medicine Berlin Germany; ^3^ Charité‐Universitätsmedizin Berlin Freie Universität Berlin Humboldt‐Universität zu Berlin Berlin Institute of Health Berlin Germany; ^4^ Max Delbrück Center for Molecular Medicine Helmholtz Association Berlin Germany; ^5^ Clinic for Pediatric Allergy, Experimental and Clinical Research Center Charité – Universitätsmedizin Berlin Freie Universität Berlin and Humboldt‐Universität zu Berlin Berlin Germany; ^6^ Si‐M/“Der Simulierte Mensch” a Science Framework of Technische Universität Berlin and Charité – Universitätsmedizin Berlin Berlin Germany; ^7^ Regenerative Immunology and Aging BIH Center for Regenerative Therapies Charité ‐ Universitätsmedizin Berlin Corporate Member of Freie Universität Berlin and Humboldt‐Universität zu Berlin Berlin Germany; ^8^ Institute of Allergology IFA Charité‐Universitätsmedizin Berlin Freie Universität Berlin Humboldt‐Universität zu Berlin Berlin Institute of Health Berlin Germany; ^9^ Fraunhofer Institute for Translational Medicine and Pharmacology ITMP Allergology and Immunology AI Berlin Germany; ^10^ Department of Biology, Chemistry and Pharmacy Freie Universität Berlin Berlin Germany; ^11^ Institute of Physiology Charité – Universitätsmedizin Berlin Freie Universität Berlin Humboldt‐Universität zu Berlin Berlin Institute of Health Berlin Germany; ^12^ Mucosal and Developmental Immunology German Rheuma Research Center Berlin (DRFZ) Berlin Germany; ^13^ Department of Microbiology, Infectious Diseases, and Immunology Laboratory of Innate Immunity Charité – Universitätsmedizin Berlin Campus Benjamin Franklin Berlin Germany; ^14^ Department of Pediatric Respiratory Medicine, Immunology and Critical Care Medicine Charité – Universitätsmedizin Berlin Freie Universität Berlin and Humboldt‐Universität zu Berlin Berlin Germany; ^15^ Institute of Social Medicine, Epidemiology and Health Economics Charité—Universitätsmedizin Berlin Freie Universität Berlin and Humboldt‐Universität zu Berlin Berlin Germany; ^16^ Department of Dermatology and Venerology Kepler University Hospital Linz Austria; ^17^ KFO339, FOOD@ Berlin Germany

**Keywords:** biomarker, epigenetics, food allergy, microbiome, peanut allergy

## Abstract

**Background:**

Peanut allergy is a frequent cause of food allergy and potentially life‐threatening. Within this interdisciplinary research approach, we aim to unravel the complex mechanisms of peanut allergy. As a first step were applied in an exploratory manner the analysis of peanut allergic versus non‐allergic controls.

**Methods:**

Biosamples were studied regarding DNA methylation signatures, gut microbiome, adaptive and innate immune cell populations, soluble signaling molecules and allergen‐reactive antibody specificities. We applied a scalable systems medicine computational workflow to the assembled data.

**Results:**

We identified combined cellular and soluble biomarker signatures that stratify donors into peanut‐allergic and non‐allergic with high specificity. DNA methylation profiling revealed various genes of interest and stool microbiota differences in bacteria abundances.

**Conclusion:**

By extending our findings to a larger set of patients (e.g., children vs. adults), we will establish predictors for food allergy and tolerance and translate these as for example, indicators for interventional studies.

## 1 INTRODUCTION

Food allergy is a major health problem and recent data indicates an increased hospitalization rate for children suffering from food‐induced anaphylaxis.^1^ Peanut is one of the most common food allergens in children and adults.^2^ Although in vivo and in vitro diagnosis of food allergy has improved in recent years, there is still the need to perform time‐consuming and potentially dangerous double‐blind placebo‐controlled food challenges to confirm the diagnosis.^3^ Moreover, it is not known which patients may be prone to develop tolerance either spontaneously or via immunotherapy protocols. Thus, molecular markers for food allergy diagnosis and response to therapy are urgently needed. Such markers may include epigenetic (e.g., IFNγ promoter),^4^, ^5^, ^6^, ^7^ microbial (e.g., prevotella),^8^, ^9^, ^10^ cellular (e.g., T cells)^11^, ^12^ and soluble factors (e.g., tryptase, PGF2).^13^, ^14^, ^15^, ^16^

We demonstrate how a multidisciplinary approach can constitute a conceptual advance to study food allergy from an integrated disease model perspective.

## 2 MATERIALS AND METHODS

### 2.1 Participants and ethics

Eligible participants were part of the randomized clinical trial TINA (Tolerance induction through non‐avoidance to prevent persistent food allergy^17^; Trial‐ID: DRKS00016764), which is currently conducted at the Charité ‐ Universitätsmedizin Berlin, Germany. The study was approved by the local ethics committee (Charité – Universitätsmedizin Berlin) (EA2/033/19). Participants were recruited in accordance with the principles of the Declaration of Helsinki from the Division of Allergy and Immunology, Department of Dermatology, Venereology and Allergy, Campus Mitte, Berlin, Germany. Subjects were ≥18 years old, with a known peanut allergy (allergic) or no food allergy (non‐allergic). Subject baseline characteristics are summarized in Supplementary Table S1. All peanut‐allergic subjects experienced at least one severe allergic reactions previously and during the oral food challenge conducted in this study. The severity grade of the reaction was classified by using the modified Sampson Score and is given in Table S1. Having sIgE to Ara h 2 was a prerequisite for the peanut‐allergic subjects. For both groups, subjects with severe concomitant diseases (e.g., cardiac) or (auto)immune diseases especially affecting the digestive tract were defined exclusion criteria.

### 2.2 Sampling and sample distribution

Biosamples were collected (Supplementary Table S2) from each subject. Fresh blood samples were subjected to basophil activation test (BAT), basophil phenotyping or PBMC. Serum was aliquoted and stored at −80°C until further use (mast cell activation test (MAT) and soluble biomarkers).

Stool samples were collected using the OMNIgene GUT kit (OM‐200, DNA Genotek, Ottawa, Canada). Samples were stored at room temperature for a maximum of seven days before storage at −80°C.

A detailed description of the other used methods are provided in the Supplementary  materials and methods section.

### 2.3 Data processing and statistical analysis

Clinical data was collected and managed using REDCap electronic data capture tools hosted at Charité.^17^ Statistical analysis is described in the specific method part above except of MAT and BAT data which were analyzed using GraphPad Prism version 9.0. In descriptive statistics, values area given as median with interquartile range (IQR). For group comparison, Mann‐Whitney test was used. Correlation coefficients were reported as *r*.

Integrative analysis of the different sub‐project data sets was performed using the R‐package metadeconfoundR (available on GitHub: https://github.com/TillBirkner/metadeconfoundR). metadeconfoundR reported significant univariate associations between individual features of all available feature spaces based on Mann‐Whitney *U*/Spearman Correlation tests (FDR‐corrected *p*‐values < 0.1). Significant associations were plotted using the R‐package circlize (v 0.4.15^18^).

## 3 RESULTS

We identified oral challenge‐proven six peanut allergic (*n* = 6) and non‐allergic (*n* = 7) individuals (details are given in Supplementary Table S1), who were interested to participate in the TINA study^17^ or to serve as a control and provided biosamples from these patients for the different analysis (Supplementary Table S2).

### 3.1 Epigenetic markers in peanut allergy

**FIGURE 1:**
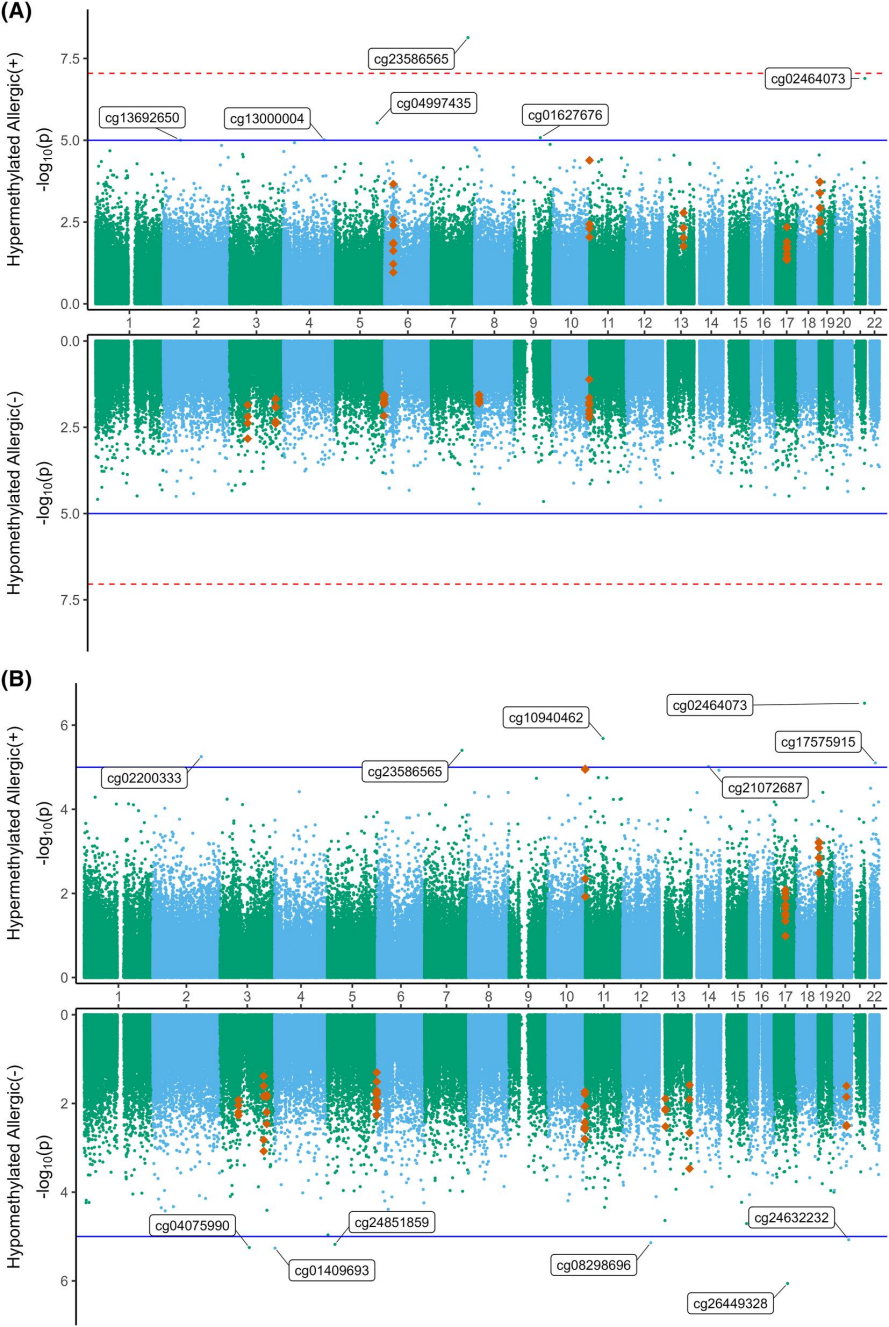
Miami plots of the methylome‐wide association analysis. Data are comparing non‐allergic and allergic phenotypes from PBMCs under (A) non‐stimulated or (B) peanut‐stimulated conditions. CpG probes are represented by green and blue dots, CpGs within differentially methylated regions (DMRs) by orange diamonds. Chromosomes are labelled 1–22 in the center of each plot. X and Y probes are excluded. The dotted red lines indicate a genome‐wide threshold of 9E‐8. Solid blue lines indicate a suggestive line of significance of 1E‐5, where potential candidate CpGs are examined. CpGs *p* < 1E‐5 are annotated. CpGs in the upper half of each plot are hypermethylated in allergic individuals (+) while in the lower half of each plot, CpGs are hypomethylated in allergic individuals (−)

The DNA methylation analysis was performed on two levels. First, we tested each individual DNA methylation probe for association with allergy status. We found a single probe, cg23586565, located in an intergenic region on chromosome 7, to be associated with allergy at genome‐wide significance (*p* = 7.29E‐9) (Figure 1A,B). Probe cg23586565 was significantly hypermethylated in allergic individuals prior to antigen stimulation (Figure 1A, Supplementary Table S3) and showed consistent suggestive (*p* < 1E‐5) hypermethylation in the same direction following stimulation (Figure 1B, Supplementary Table S3). Probe cg0246073, located on chromosome 21 within the promoter region of *ITGB2*, remained similarly hypermethylated in allergic individuals both prior and post stimulation (Supplementary Table S3). However, we found differences in the overall landscape of detected candidate probes in stimulated and non‐stimulated PBMCs, with more candidate probes appearing after antigen stimulation. Secondly, as DNA methylation typically occurs across multiple CpG islands within a regulatory region, we analyzed the methylation status of multiple CpG islands to identify differentially methylated regions (DMRs). The results are summarized in Figure 1. A total of 21 DMRs were detected at genome‐wide significance comparing allergic versus non‐allergic individuals, most of which were located within gene promoters. The top DMR associations include regions overlapping genes *SMIM24*, *SCART1*, and *RUFY1* (sidak‐*p* < 0.05). A full list of candidate CpGs is presented in (Supplementary Table S3).

### 3.2 Gut microbial composition in peanut‐allergic patients

**FIGURE 2:**
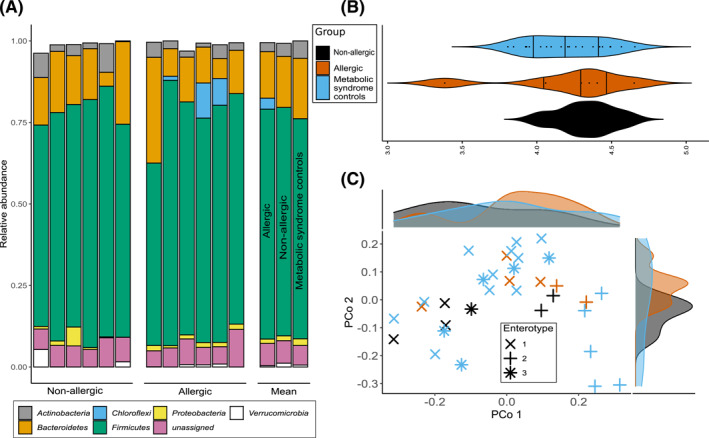
Gut‐microbial composition of study samples (“non‐allergic” and “allergic”) in comparison to samples from Maifeld et al.^19^ (A) Relative abundance of most abundant phyla for each individual sample (left, middle) and mean per group including metabolic syndrome samples (right). (B) Violin plot of alpha diversity based on Shannon index; vertical lines indicate quartiles; color indicates sample group (C) principle coordinate analysis of beta‐diversity based on bray‐curtis distance; color indicates patient group, symbol shape indicates enterotype assignment based on Dirichlet‐Multinomial Mixture Model. Marginal density plots show distribution of sample groups along PCo1 and PCo2

The relative abundance of gut bacterial phyla did not differ strongly between allergic, non‐allergic, or control subjects, though abundance of Chloroflexi differs significantly between these three groups (Kruskal Wallis test FDR: 0.03) (Figure 2A). No significant differences in alpha diversity based on the Shannon index were found between these groups (Figure 2B). Beta diversity analysis based on Bray‐Curtis dissimilarity does not reveal significant overall gut microbiome compositional differences between the tested groups (Figure 2C) in our pilot study dataset, and in line with this, enterotype distribution between the groups likewise is not significantly different (Figure 2C) at the present sample size.

### 3.3 Increased T cell functional diversity in peanut‐allergic individuals

**FIGURE 3:**
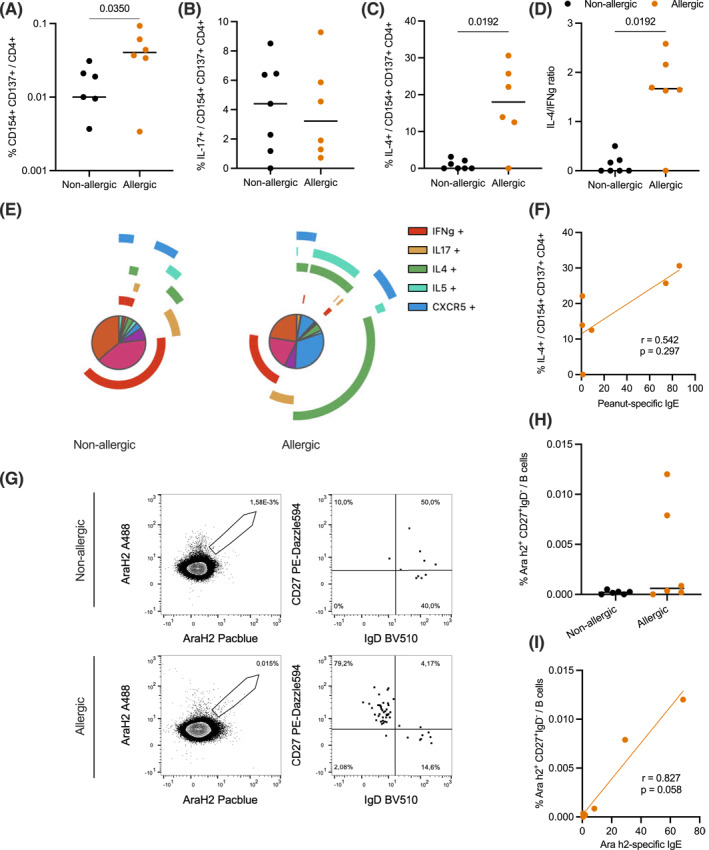
Frequency and function of allergen‐specific T cells in non‐allergic and allergic subjects. (A–D) Allergen‐specific T cells (CD154^+^ CD137^+^) in CD4^+^, and their IL‐17, IL‐4 production and IL4/IFNg ratio in non‐allergics and allergics. (E) Polyfunctionality analysis comparison of reactive CD4s after stimulation with peanut extract. Arcs are indicating expression of IFNg (red), IL‐17 (yellow), IL‐4 (green), IL‐5 (turquoise) and CXCR5 (blue), arranged in this order from inside to outside. Colors in the pie chart indicate different subpopulations, categorized based on cytokine and marker expression (legend displayed in Supplementary Figure3B). (F) Correlation of IL‐4^+^ peanut‐specific CD4^+^ T cells and peanut‐specific IgE in allergic donors (Spearman correlation). (G) Ara h2‐specific B cells staining in a non‐allergic and allergic donor. (H) Ara h2‐specific memory class‐switched (CD27^+^ IgD^−^) B cells in non‐allergic versus allergic donors. (I) Correlation of Ara h2‐specific memory (CD27^+^ IgD^−^) B cells and Ara h2‐specific IgE in allergic donors. Statistical comparison were performed using the Mann‐Whitney test

In allergic donors, peanut‐specific helper T cells were 4‐times more abundant and exhibited stronger IL‐4 production and a higher IL‐4 to IFNγ ratio, indicative of a Th2 phenotype, while in non‐allergic donors, a dominant Th1 phenotype (producing IFNγ) was observed (Figure 3A–E). Regarding poly‐functionality, allergic donors displayed higher diversity with more Th2 and Tfh phenotypes within allergen‐specific T cells (Figure 3E). We observed no significant difference in allergic donors regarding the frequency of IL‐17 production. The frequency of IL‐4‐producing allergen‐reactive CD4^+^ T cells showed a tendency to correlate with peanut‐specific IgE (Figure 3F).

### 3.4 Detection of allergen‐specific B cells and their correlation to specific IgE responses

Dual labeling of Ara h 2 with two fluorochromes (Figure 3G) was performed to identify allergen‐specific B cells. Using this strategy, a subset of peanut‐allergic donors (two of six) had a 3‐fold higher proportion of circulating Ara h2‐specific memory class‐switched B cells (defined as CD27^+^ IgD^−^, Figure 3G–H) than non‐allergic donors. Moreover, their frequency correlated with the serum titers of Ara h 2‐specific IgE (Figure 3I).

### 3.5 BAT and MAT in peanut‐allergic patients

**FIGURE 4:**
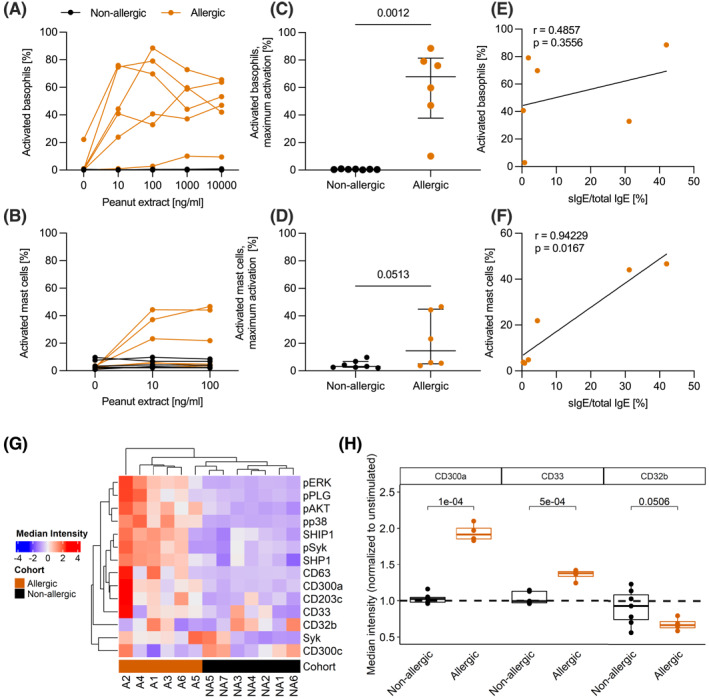
Peanut induced activation of basophils and PSCMCs sensitized with serum of non‐allergic and allergic individuals as well as basophils response to peanut allergen in peanut‐allergic and non‐allergic donors. (A) BAT: Percentage of CD63^+^ basophils to increasing dose of peanut extract in non‐allergic and allergic donors (B) MAT: Percentage of CD63^+^ PSCMCs, sensitized with serum from non‐allergic and allergic individuals to increasing dose of peanut extract (C) The maximal basophil activation is significantly different between non‐allergic and allergic donors. (D) The maximal mast cell activation is different between non‐allergic and allergic donors (E) Correlation of percentage of activated basophils after stimulation with 100ng/ml peanut extract with the sIgE/total IgE ratio. (F) Correlation of percentage of activated mast cells after stimulation with 100ng/ml peanut extract with the sIgE/total IgE ratio. (G) Basophils phenotyping: Whole blood was stimulated with 1 μg/ml of peanut extract for 5 min or left unstimulated, and the basophil phenotyping was conducted using mass cytometry. Heatmap depicting clustering of the allergic and non‐allergic donors based on different markers expression in the basophils. (H) Boxplot representing normalized expression to unstimulated of three inhibitory receptors (CD300a, CD33 and CD32b) in the basophils after stimulation with peanut extract. Descriptive statistics are represented as median with interquartile range and Mann‐Whitney test was used for comparison. Correlations were determined using non‐parametric Spearman correlation. *p*‐values <0.05 were determined as significant

Next we compared the basophil and the mast cell activation test (BAT and MAT) regarding their sensitivity towards allergens. Patient basophils as well as passively sensitized peripheral CD34^+^ stem cell‐derived mast cells (PSCMCs) were stimulated with peanut extract and their activation was assessed by CD63 surface staining. For both tests, allergen stimulation resulted in a dose‐dependent activation, but not the controls (Figure 4A,B). However, both cohorts (non‐allergic and allergic) exhibited mast cell and basophil activation after anti‐IgE stimulation (data not shown). Maximal basophil and mast cell activation differed between non‐allergic and allergic donors. Although both tests discriminated between allergic and non‐allergic, only three of six allergic patients exhibited mast cell activation (Figure 4C,D). Consecutively, we suspected that the mast cell activation test is dependent on specific IgE levels in the serum sample. Indeed, the mast cell‐ but not basophil‐activation after stimulation with 100 ng/ml peanut extract correlated with the sIgE/total IgE ratio (Figure 4E,F).

### 3.6 Differential expression of inhibitory receptors on basophils as novel stratification markers

To deeply characterize basophils, we used mass cytometry to measure a multitude of basophil markers such as inhibitory receptors or phosphorylation of signaling proteins downstream of the high‐affinity IgE receptor (FcεR1). After stimulation with a peanut extract, we observed differential expression between allergic and non‐allergic donors of activation markers (CD63, CD203c), inhibitory receptors (CD32b, CD300a, CD33) and phosphorylation of signaling proteins (pp38, pAKT, pSyk) in the basophils (Figure 4G). Furthermore, we determined a modified expression of inhibitory receptors of the IgE‐mediated signaling pathway in allergic but not in non‐allergic donors: CD300a and CD33 expression was increased at the surface of the basophils, while CD32b expression was decreased (Figure 4H). Hence, clear differences in basophil phenotypes after allergen stimulation were observed between allergic and non‐allergic donors.

### 3.7 Novel serological biomarkers in peanut allergy

**FIGURE 5:**
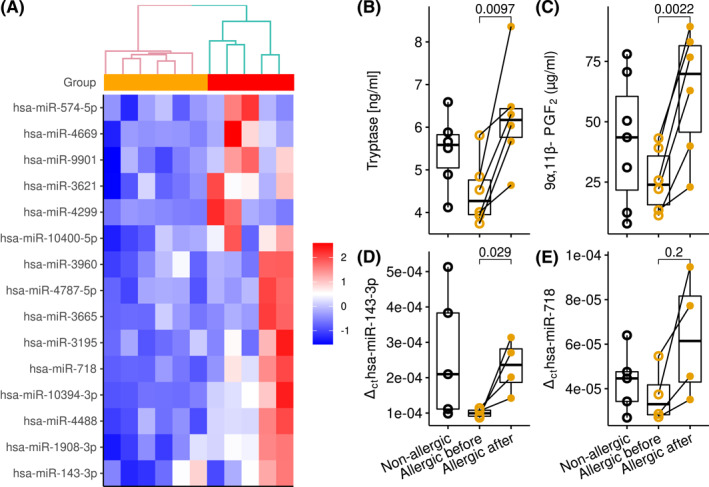
Serological biomarkers upon oral food challenge. (A) Heatmap of 15 most differentially expressed miRNAs in peanut‐allergic patients undergoing oral food challenge (measured 1 hour after reaction)—orange and before oral food challenge—brown; (B, C) Concentration of protein biomarkers in non‐allergic (*n* = 6) and allergic (*n* = 6) individuals; (D, E) Expression of selected miRNA biomarkers in serum of non‐allergic (*n* = 5) and allergic individuals (*n* = 4), validated by RT‐qPCR. Only four and five samples could be analyzed in qPCR data as remaining samples were hemolyzed as it affects miRNA levels in serum due to overspill of the intracellular compartment into extracellular fluid^20^

To identify serological biomarkers of peanut allergy during oral food challenge, we measured known biomarkers such as tryptase and PGF2 but also miRNAs. For this analysis blood samples were taken 1 hour after positive oral food challenge. Tryptase was elevated in six out of six peanut‐allergic patients (Figure 5B). The baseline value of the same individual was required to detect this elevation due to the pronounced inter‐individual variability.^21^ Similar findings were observed for PGF2 (Figure 5C), which has been reported by us previously as a biomarker of real‐life anaphylaxis.^13^, ^14^ Other analytes measured were Apolipoprotein (Apo)A1, ApoE, arachidonic acid and eosinophil cationic protein (ECP)^15^ (not shown). ApoA1 was reported to be downregulated during anaphylaxis, also trending lower upon peanut challenge without reaching significance in this dataset (not shown).

Next‐generation sequencing allowed for a good separation of samples originating from peanut‐allergic patients before versus timepoint of positive oral food challenge with peanuts, suggesting allergen‐triggered changes in miRNA patterns. Figure 5A illustrates 15 most differentially expressed (upregulated) miRNA. Results from sequencing were validated by qPCR on an independent sample set. hsa‐miR‐143‐3p and hsa‐miR‐718 increased upon oral food challenge in peanut‐allergic patients while showing a relatively large variability within the non‐allergic adults (Figure 5D,E).

### 3.8 Intercorrelating results from multiple‐omics analyses

**FIGURE 6:**
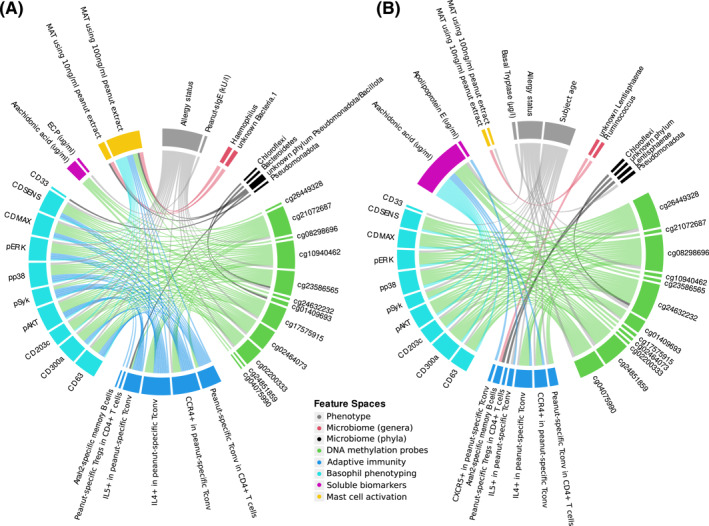
Integrative circos plot showing significant positive (A) and negative (B) associations between individual features measured by the FOOD@ consortium. Each line between two features shows a significant association (Mann‐Whitney *U*/Spearman Correlation test, FDR‐corrected *p*‐values <0.1). The line thickness indicates the effect size (Cliff's Delta/Spearman's rho) of the respective association. The outer ring segments show corresponding feature space (color) and number/strength of associations for each feature. Detailed legend displayed in Supplementary Table S4. Median fluorescence intensity are given for the basophil markers pERK, pp38, pSyk, pAKT, CD203c, CD300a, CD63 and CD33, CDMAX—Percentage of maximum of activated basophils, CDSENS—peanut extract concentration in ng/ml required for activation of 50% of the responsive basophils. T and B cell subsets are given in percentage. ECP, eosinophil cationic protein; MAT, mast cell activation test; Tconv, conventional T cells; Treg, regulatory T cells

Finally, we integrated our data from the separate experimental approaches to assess whether any overarching patterns become visible. The findings reported from each discipline are shown together with their intercorrelations as a circos plot (Figure 6). Although no definitive conclusions can be drawn due to the small sample size, this analysis revealed multiple feature space intercorrelations of signals from defined microbiota, epigenetic loci, cellular and soluble biomarkers as well as mast cell activation within our cohort.

## 4 DISCUSSION

Here, we present an interdisciplinary approach to delineate novel approaches for a better understanding of food allergy. Although at this stage, some of the data remain preliminary for any definitive conclusions (e.g., microbial and epigenetic data) to be drawn, we could confirm previous research findings for example, regarding the BAT and MAT but also identified new biomarkers, which are currently validated in a larger cohort.

For the methylation analysis we found a strong genome‐wide association between phenotypes from probe cg23586565, with closest protein‐coding genes *FAM180A* and *MTPN* (Figure 1 and Supplementary Table S3; *p* = 7.29E‐09). We observed a potential interaction between this gene and the open chromatin region in which cg23586565 is located, which may indicate this region as a regulator of *FAM180A* expression (Supplementary Figure S1).^22^, ^23^ Our detected DMRs mostly overlap across analyses, indicating independence to antigen stimulation, and were shown to be primarily involved in cell cycle and homologous recombination pathways (Supplementary Table S3; Supplementary FigureS2). In this study we identified two DMRs which are overlapping *RUFY1* and *SCART1*. These regions are also implicated in infant food‐ and aero‐allergen sIgE outcome in a previous study with children at 5 years of age.^24^ *RUFY1*, in context of aero‐allergens, presented in a different methylation direction between the studies, while *SCART1*, in context of food allergy, did not. This might point to a differing role of aero‐ and food‐allergen responses. Overall, we report strong DNAm signals associated with peanut allergy in adults at a genome‐wide level, with DMR signals replicating in an independent study. The incomplete overlap in detected genes with and without antigen‐stimulation, respectively, indicates that some DNAm signals are stable and act independently of stimulation, while other signals appear to be dependent on stimulation.

Regarding the microbiome, we show that the gut microbial composition of subjects with food allergy on an overall scale is comparable to subjects without food allergy and to patients with metabolic syndrome,^19^ with no gross alterations, though a subtler signature especially of this cannot be excluded and even may be likely to be confirmed once higher‐powered cohorts are available. Even given the explorative nature of the current study, we could identify the phylum Chloroflexi to be significantly enriched in food allergic subjects. This phylum is unexplored, with most of its representatives being uncultivatable and found in diverse environments, including the human oral cavity.^25^ More interestingly, Chloroflexi has recently been associated with allergic rhinitis as a unique phylum in allergic rhinitis subjects.^26^ Further study with larger sample size, as we are planning, should verify this preliminary finding in subjects with food allergies.

At the cellular level allergic and non‐allergic donors were clearly separable according to the frequency of peanut extract reactive CD4^+^ T cells, their IL‐4 and IL‐5 expression, as well as the presence of CXCR5^+^ Tfh cells. This is in accordance with previous studies.^11^ Moreover, we observed positive correlations of IL‐4 production and titers of peanut‐specific IgE as well as Ara h 2‐specific B cells and Ara h 2‐specific IgE. IL‐17 production was not significantly different between the groups, which does not support previous reports about increased Th17 frequencies in some allergic donors.^27^ However, a larger sample size will be required to further identify Ara h 2‐specific B cell signatures and to characterize its relation to T cell signatures.

As the use of basophils from peripheral blood has some limitations the MAT has been suggested.^28^, ^29^ Here we have tested the usefulness of the MAT in patients with peanut allergy in direct comparison with the BAT. Both tests are able to discriminate between non‐allergic and allergic patients with the BAT being superior to the MAT. However, the MAT has the advantage of using stored frozen serum samples, which are not time‐sensitive for testing. In three patients, who had sIgE levels lower than 1.5 kU/L, none or only very low mast cell activation was observed after stimulation with antigen extract, suggesting that the MAT is only useful in patients that have sIgE levels above a certain threshold.

The characterization of the innate immune system response of peanut‐allergic and non‐allergic donors to peanut extract stimulation revealed activation only of basophils from allergic donors with modified expression of inhibitory receptors of the IgE‐mediated signaling pathway: CD300a and CD33 expression was increased at the surface of the basophils, while CD32b expression was decreased. The increase in CD300a and CD33 may indicate a refractory state after degranulation, since both markers have been associated with inhibited degranulation or mast cell desensitization.^30^, ^31^ The downregulation of CD32b at the surface of the basophils could be due to a release of this receptor, which once bound by allergen‐specific IgG, could act as a decoy.^32^ We hypothesize that these three regulatory receptors may be involved in basophil anergy induced by repeated allergen challenge, and therefore, could form promising targets for the stratification of donors and prediction of intervention success.

The miRNAome sequencing was recently uncovered as a promising approach to reveal informative biomarkers in the context of anaphylaxis.^15^ We used a similar strategy to uncover viable candidates in oral food challenge. In fact, miRNA sequencing revealed several altered entities, of which two were selected for further analysis. Sequencing results could be verified by qPCR, providing independent validation. The miR‐143‐3p was increased during oral food challenge, while miR‐718 showed a tendency, indicating that both entities will turn out suitable in the significantly expanded cohorts. Interestingly, while miR‐143‐3p is abundantly expressed by many cells, miR‐718 was below detection across the entire Fantom5 miRNA Atlas encompassing 492 short RNA libraries, suggesting a very limited expression range.^33^ Collectively, serum miRNAs show potential as easily obtainable, sensitive, and fast‐reacting biomarkers of oral food challenges.

Finally, the system‐wide analysis of the presented multi‐omics assessments revealed some promising correlations linking diverse epigenetic, cellular, serological and clinical parameters, which may allow for identification of so far unknown physiological mechanisms. However, the present dataset is limited in statistical power for a comprehensive analysis. Further expansion of the dataset will scale within this approach, allowing easy assessment of whether multiple parallel indicators of a food allergy status also can be shown to be independently linked (e.g., shown correlating under stratification for food allergy status or for intervention). This will then enable us to form candidate chains of effect for further validation of putative mechanisms as well as to identify candidate intervention targets. Their clinical utility and specificity must be further validated in larger cohorts.

Taken together, our data suggest epigenetic patterns distinguishing in patients with peanut allergy, which may be linked to known and novel immunological cellular and soluble biomarkers as well as possibly defined microbiota. By applying detailed clinical characterization, high‐throughput measurements of serological, microbial and epigenetic phenotypes and bioinformatic data integration we now have established a multi‐omics analysis flow that we will apply to larger well‐defined patient cohorts (e.g., children vs. adults) in order to identify, characterize, validate and report further predictors for peanut allergy and tolerance as well as for indications of the use of various treatments.

## AUTHOR CONTRIBUTIONS

**Margitta Worm**: Conceptualization (Lead); Data curation (Equal); Formal analysis (Equal); Funding acquisition (Lead); Investigation (Lead); Methodology (Equal); Project administration (Lead); Resources (Lead); Supervision (Lead); Validation (Lead); Visualization (Lead); Writing – original draft (Lead); Writing – review & editing (Lead). **Aikaterina Alexiou**: Data curation (Supporting); Investigation (Supporting); Project administration (Supporting); Writing – review & editing (Supporting). **Veronika Hofer**: Data curation (Supporting); Investigation (Supporting); Project administration (Supporting); Writing – review & editing (Supporting). **Till Birkner**: Data curation (Supporting); Formal analysis (Supporting); Investigation (Supporting); (Supporting); Methodology (Supporting); Software (Equal); Validation (Supporting); Visualization (Equal); (Equal); Writing – review & editing (Supporting). **Alexander C. S. N. Jeanrenaud**: Data curation (Supporting); Formal analysis (Supporting); Investigation (Supporting); Methodology (Supporting); Software (Equal); Validation (Supporting); Visualization (Equal); Writing – review & editing (Supporting). **Florent Fauchere**: Data curation (Supporting); Formal analysis (Supporting); Investigation (Supporting); Methodology (Supporting); Software (Equal); Validation (Supporting); Visualization (Supporting); Writing – review & editing (Supporting). **Kristijan Pazur**: Formal analysis (Supporting); Investigation (Supporting); Methodology (Supporting); Visualization (Supporting); Writing – review & editing (Supporting). **Carolin Steinert**: Formal analysis (Supporting); Investigation (Supporting); Methodology (Supporting); Validation (Supporting); Visualization (Supporting); Writing – review & editing (Supporting). **Aleix Arnau‐Soler**: Formal analysis (Equal); Methodology (Supporting); Supervision (Equal); Validation (Equal); Visualization (Equal); Writing – review & editing (Supporting). **Priyanka Banerjee**: Resources (Supporting); Supervision (Supporting); Writing – review & editing (Supporting). **Andreas Diefenbach**: Funding acquisition (Supporting); Resources (Supporting); Writing – review & editing (Supporting). **Josefine Dobbertin‐Welsch**: Investigation (Supporting); Resources (Supporting); Writing – review & editing (Supporting). **Sabine Dolle‐Bierke**: Data curation (Supporting); Investigation (Supporting); Project administration (Supporting); Supervision (Supporting); Visualization (Supporting); Writing – original draft (Supporting); Writing – review & editing (Supporting). **Wojciech Francuzik**: Data curation (Supporting); Formal analysis (Supporting); Investigation (Supporting); Methodology (Supporting); Software (Equal); Validation (Supporting); Visualization (Supporting); Writing – review & editing (Supporting). **Ahla Ghauri**: Methodology (Supporting); Writing – review & editing (Supporting). **Stephanie Heller**: Methodology (Supporting); Writing – review & editing (Supporting). **Birgit Kalb**: Investigation (Supporting); Writing – review & editing (Supporting). **Ulrike Lober**: Formal analysis (Supporting); Methodology (Supporting); Software (Supporting); Visualization (Supporting); Writing – review & editing (Supporting). **Ingo Marenholz**: Data curation (Supporting); Formal analysis (Supporting); Funding acquisition (Supporting); Investigation (Equal); Methodology (Equal); Resources (Supporting); Supervision (Supporting); Validation (Supporting); Visualization (Supporting); Writing – review & editing (Supporting); Lajos Marko: Methodology (Supporting); Writing – review & editing (Supporting). **Jorg Scheffel**: Methodology (Supporting); Supervision (Supporting); Writing – review & editing (Supporting). **Olena Potapenko**: Methodology (Supporting); Writing – review & editing (Supporting). **Stephanie Roll**: Data curation (Supporting); Formal analysis (Supporting); Funding acquisition (Supporting); Resources (Supporting); Supervision (Supporting); Writing – review & editing (Supporting). **Susanne Lau**: Data curation (Supporting); Formal analysis (Supporting); Funding acquisition (Supporting); Methodology (Supporting); Resources (Supporting); Supervision (Supporting); Writing – review & editing (Supporting). **Young‐Ae Lee**: Data curation (Supporting); Formal analysis (Supporting); Funding acquisition (Equal); Investigation (Equal); Methodology (Equal); Resources (Supporting); Software (Supporting); Supervision (Equal); Validation (Equal); Visualization (Equal); Writing – review & editing (Supporting). **Julian Braun**: Data curation (Supporting); Formal analysis (Supporting); Investigation (Supporting); Methodology (Equal); Software (Equal); Supervision (Supporting); Validation (Supporting); Visualization (Equal); Writing – review & editing (Supporting). **Andreas Thiel**: Data curation (Supporting); Formal analysis (Supporting); Funding acquisition (Supporting); Methodology (Equal); (Equal); Resources (Supporting); Supervision (Supporting); Writing – review & editing (Supporting). **Magda Babina**: Data curation (Supporting); Formal analysis (Supporting); Funding acquisition (Equal); Investigation (Equal); Methodology (Equal); Resources (Supporting); Supervision (Supporting); Validation (Supporting); Visualization (Supporting); Writing – review & editing (Supporting). **Sabine Altrichter**: Data curation (Supporting); Formal analysis (Supporting); Funding acquisition (Equal); Investigation (Equal); Methodology (Equal); Resources (Supporting); Supervision (Supporting); Validation (Supporting); Writing – review & editing (Supporting). **Sofia Forslund**: Data curation (Supporting); Formal analysis (Supporting); Funding acquisition (Equal); Investigation (Equal); Methodology (Equal); Resources (Supporting); Software (Equal); Supervision (Supporting); Validation (Supporting); Visualization (Supporting); Writing – review & editing (Supporting). **Kirsten Beyer**: Conceptualization (Lead); Data curation (Supporting); Formal analysis (Supporting); Funding acquisition (Lead); (Lead); Investigation (Lead); Methodology (Equal); Project administration (Lead); Resources (Lead); Supervision (Lead); Validation (Supporting); Visualization (Equal); Writing – original draft (Supporting); Writing – review & editing; Equal.

## CONFLICT OF INTEREST

All authors declare no competing interests.

## Supporting information

Supplementary Material S1
