# Supplementary material for: An interdisciplinary approach to characterize peanut‐allergic patients—First data from the FOOD@ consortium
Source: Clin Transl Allergy. 2022 Oct 5;12(10):e12197. doi: 10.1002/clt2.12197 (PMC9533219; doi:10.1002/clt2.12197)
Supplement: Supplementary file 1 — Supplementary Material S1 [file CLT2-12-e12197-s001.docx]

**Supplementary Material&Methods, Tables and Figures**

**Supplementary Method and Methods section**

**Generation and quality control of peanut extract**

*Peanut extract*: The allergen extract was prepared using unpeeled peanuts (type Virginia, Kaufland, Germany). Paste-like flour was produced by using a universal shredder system. The flour was degreased with acetone (Merch Millipore, SupreSolv) for 2 h prior to filtering (Machery Nagel, MN 615) and overnight drying at room temperature. The dried powder was solved in PBS-buffer (Corning) containing protease inhibitor (Roche) until a homogenous solution was reached. This solution was mixed overnight at 4 ^o^C and centrifuged (2300 x g, 20 min). The upper greasy film was discarded. Peanut extract was stored in aliquots at -20 ^o^C until further use.

*Protein and endotoxin estimation*: Protein concentration was estimated using the Pierce^TM^ BCA Protein Assay (23225, ThermoSci). Protein stock concentration was estimated to be 13.8 mg/ml. Endotoxin quantification was performed using the Pierce^TM^ Chromogenic Endotoxin Quant assay (A39553, ThermoSci). Stock protein endotoxin was estimated to be 46.6 EU/ml. Following dilution of the peanut extract to 50 µg/ml for cell culture application, endotoxin was 0.17 EU/ml.

*SDS and Immunoblot*: To confirm the presence of specific proteins, SDS-PAGE (1 µg protein/lane, NuPage 4-12% Bis-Tris gel, Invitrogen) and immunoblot assays were performed. Serum pools with high concentrations of peanut-specific IgEs were used as primary detection antibodies. Atopic-positive and -negative serum pools were used as controls. The secondary detection was performed with an antibody complex (goat anti-human IgE/mouse anti-DIG-HRP, DST).

**DNA isolation from PBMCs**

Frozen-thawed PBMCs were resuspended in 500 µl RPMI 1640-Medium (containing L-Glutamine; Gibco) and supplemented with 10% human AB serum (Gemcell). PBMCs were cultured at a density of 4x10^6^/ml with both 50 µg/ml peanut allergen extract and no stimulation per sample for 48 h at 37 °C, 5% CO_2_. Cultured PBMCs were resuspended in RLT buffer (Qiagen). DNA and RNA were separated using a Qiagen DNA column (Qiagen). DNA concentration and quality was assessed using a NanoDrop.

**DNA methylation analysis**

For methylation profiling, 500 ng of genomic DNA were treated with sodium bisulfite per sample using the Infinium Methylation EPIC BeadChip (Illumina) on a HiScan scanner (Illumina) according to the manufacturer’s standard protocols. All samples were pre-processed, normalized, and analyzed using meffil.^1^ Raw intensity values were pre-processed and quantile-normalized with respect to 4 PCs. Normalization was adjusted for using slide, sentrix row, sex, and estimated cell type composition. QC thresholds of > 5% of CpGs with a detection p-value > 0.01, or a bead count < 3 were used. No samples were removed using this QC. Non-specific and sex chromosomes-associated CpGs were removed. 780113 CpGs remained for further analysis by meffil, whereafter surrogate variables were adjusted. The genome-wide significance threshold was set to 9E-8.^2^ Comb-p algorithm was used to identify DMRs by combining spatially correlated p-values from the DNAm analysis.^3^ CpGs were combined to a seed p-value of 0.001, a maximum distance of 750 bp, and a minimum of three CpGs. For DMRs, a Šidák correction for multiple testing was used to determine statistical significance at p-value < 0.05. For probes or DMRs not within or surrounding genes, the closest transcriptional start sites of the nearest protein coding genes were found using GREAT.^4^

**Pathway enrichment and interaction analysis**

Pathway enrichment was conducted using the online web tool PathwaX against pathways in KEGG v94.1.^5^ FWER q-values were reported for pathway enrichment. For chromatin and expression quantitative trait locus (eQTL) interactions of cg23586565, summary statistics of the association analysis from the non-stimulated condition were passed onto the tool “FUMA - functional mapping and annotation of genetic associations”.^6^ As cg23586565 was overlaying a region with a very rare SNP frequency, additional SNPs of interest were identified in the surrounding region and provided as part of the summary statistics into FUMA. Interactions with FDR *P* < 1E-3 and *P* < 1E-6 were considered for eQTL and chromatin interactions, respectively.

**Stool sample isolation and sequencing**

DNA isolation was conducted under a hood with laminar flow (LabGarda ES Energy Sever Classe II Laminar Flow, NuAire Inc., Plymouth, Minnesota) to limit environmental contamination. Total DNA was extracted with the ZymoBIOMICS DNA Miniprep Kit (ZYMO Research Europe GmbH, Freiburg, Germany) using the manufacturer’s instruction. Isolated DNA concentration was measured by Qubit (Thermo Fisher Scientific, Waltham, WA), DNA integrity and purity were assayed using a 5400 Fragment Analyzer (Agilent, Santa Clara, CA, United States). All samples isolated passed the quality control measures. Metagenomics library preparation and sequencing (Illumina NovaSeq 6000 PE150, at minimum of 6G raw data per sample) was performed by Novogene (Novogene (UK) Company Limited).

**Analysis of stool microbiome composition**

Study biosamples were processed together with 23 samples from an already published data set of patients with metabolic syndrome.^7^ Quality control, pairing and filtering of the sequences was performed using the NGLess pipeline (v1.3.0)^8^. Preprocessed reads were taxonomically classified using mOTUs (v2.6)^9^ with NGLess. The data analyses were performed using the R programming language (v 4.1.2). Rarefication of samples to the sampling depth of the smallest retained samples (5350 reads) as well as computation of Shannon alpha diversity index was performed using the R package rtk (v 0.2.6.1). Samples were clustered into three enterotypes using the R package DirichletMultinomial (v 1.34.0). Beta diversity was calculated as Bray-Curtis dissimilarity using the R package vegan (v 2.5-7).

**Allergen-specific T cells**

Freshly isolated PBMCs depleted from CD19+ cells were cultivated at 10x10^6^ PBMC in AB-medium containing RPMI 1640 medium (Gibco) supplemented with 10% heat-inactivated AB serum (Pan Biotech), 100 U/ml penicillin (Biochrom), 0.1 mg/ml streptomycin (Biochrom). Stimulations were conducted with peanut extract at a concentration of 50 µg/ml. Stimulation controls were performed with PBS (unstimulated control) and 1.5 mg/ml SEB and 1 mg/ml TSST1 (Sigma-Aldrich). All approaches contained 1 µg/ml purified anti-CD28 (clone CD28.2, BD Biosciences). Incubation was performed at 37 °C, 5% CO2 for 18 h in the presence of 10 µg/ml brefeldin A (Sigma-Aldrich) during the last 16 h. Stimulations were stopped by incubation in 20 mM EDTA for 5 min. Surface staining was performed for 20 min in the presence of 1 mg/ml beriglobin (CSL Behring) with the following fluorochrome-conjugated antibodies titrated to their optimal concentrations. The list of antibodies used for staining can be found in the supplementary (**Supplementary Table S5**). During the last 5 min of incubation, Zombie Yellow fixable viability staining (Biolegend) was added. Fixation and permeabilization were performed with eBioscience^TM^ FoxP3 fixation and PermBuffer (Invitrogen) according to the manufacturer’s protocol, and intracellular staining was carried out for 30 minutes. All samples were measured on a MACSQuant^®^ Analyzer 16 (Miltenyi) according to the gating strategy illustrated in **Supplementary Figure S31A**. Instrument performance was monitored before every measurement with Rainbow Calibration Particles (BD). Flow cytometry data were analyzed using FlowJo 10 (BD), R and Spice (**Supplementary Figure S3B**).

**Allergen-specific B cells**

B cells were isolated from PBMCs using CD19 microbeads (Miltenyi) and LS column (Miltenyi). Surface staining was performed for 30 min in the presence of 1 mg/ml beriglobin (CSL Behring) and with Ara-H-2 (Indoor biotechnologies) conjugated with PacificBlue or Alexa Fluor 488 (Invitrogen) according to the manufacturer’s protocol. The list of antibodies used for staining can be found in the supplementary (**Supplementary Table S6**). During the last 5 min of incubation, Zombie Yellow fixable viability staining (Biolegend) was added. Fixation and permeabilization were performed with eBioscience^TM^ FoxP3 fixation and PermBuffer (Invitrogen) according to the manufacturer’s protocol and intracellular staining was carried out. All samples were measured on a MACSQuant^®^ Analyzer 16 (Miltenyi) according to the gating strategy illustrated in **Supplementary Figure S4**.

**Basophil phenotyping**

200 µl of whole blood was incubated at 37 °C, 5% CO_2_ for 20 min. During the incubation, surface staining of selected surface markers was performed in the presence of 1 mg/ml beriglobin (CSL Behring). The list of antibodies used for staining can be found in supplementary (**Supplementary Table S7**). During the last 5 min of incubation, whole blood was stimulated with PBS (unstimulated control), 10 µg/ml anti-IgE antibodies (MHE-18, Biolegend) or with 1 µg/ml of peanut extract, and live dead staining was performed with Cell-ID Cisplatin-196Pt (Fluidigm). Stimulations were stopped and samples were fixed by incubation with Proteomic Stabilizer (Smart Tube inc) and 20nM EDTA for 10 min, and samples were cryopreserved at -80 °C using a CoolCell (BioCision). Samples were thawed and Thaw-Lyse Buffer (Smart Tube inc) was added according to the manufacturer’s protocol. Samples were barcoded and pooled together using a combination of three isotopes conjugated B2M antibody (2M2, Biolegend) out of six. All the antibodies used for mass cytometry were conjugated using X8 or MCP9 MaxPar conjugation kits (Fluidigm) according to the manufacturer's instructions. Next, pools were depleted from Neutrophils and T cells using the following biotinylated antibody: CD15 (VIMC6, Miltenyi) and CD3 (REA613, Miltenyi) together with anti-biotin Microbeads (Miltenyi) and LS columns (Miltenyi). Staining of surface markers was then performed with isotope conjugated antibody for 20 min. Afterwards, samples were fixed with 4% PFA (Invitrogen) and permeabilized with BD Phosphoflow Perm Buffer II (BD Bioscience) according to the manufacturer’s protocol. Intracellular staining was performed for 30 min in the dark and the samples were further fixed with 4% PFA overnight while DNA staining was performed with Cell-ID intercalator-Ir (Fluidigm). Samples were measured on a Helios mass cytometer (Fluidigm). Obtained data was compensated using R (CATALYST package). Debarcoding and gating of basophils illustrated in **Supplementary Figure S5** were performed with Cytobank and analysis on R.

**Basophil Activation Test**

40 µl of whole blood was incubated at 37 °C, 5% CO_2_ for 30 min with PBS (unstimulated control), 10 µg/ml anti-IgE antibodies (MHE-18, Biolegend) or with 10 ng/ml, 100 ng/ml, 1 µg/ml and 10 µg/ml of peanut extract. During the incubation, surface staining was performed in the presence of 1 mg/ml beriglobin (CSL Behring). The list of antibodies used for staining can be found in supplementary (**Supplementary Table S8**). Stimulations were stopped and samples were fixed by incubation with 20nM EDTA and BD FACS lysing solution (BD Biosciences) for 10 min. Staining post-fixation was performed and the samples were permeabilized with BD Phosphoflow Perm Buffer II (BD Bioscience) according to the manufacturer’s protocol. Intracellular staining was carried out for 30 min in the dark at room temperature. All samples were measured on a MACSQuant^®^ Analyzer 16 (Miltenyi) according to the gating strategy illustrated in **Supplementary Figure S6**. Instrument performance was monitored before every measurement with Rainbow Calibration Particles (BD). Flow cytometry data were analyzed using FlowJo 10 (BD).

**Soluble biomarkers**

Human serum samples were analyzed using ELISA kits for 11β- Prostaglandin F2α, (Cayman Chemical Company, Ann Arbor, USA, 516521) and Human Apolipoprotein A1 (Abcam, Cambridge, UK, ab108804). Total concentration of tryptase in serum was measured by ImmunoCAP (Thermo Fisher Scientific, Waltham, USA).

For sequencing and qPCR quantification of miRNAs total RNA was isolated from 200 µl serum by the miRNeasy Serum/Plasma kit (Qiagen, Hilden, Germany, 217184) according to the manufacturer’s instructions. Library preparation was performed using QIASeq miRNA library kit (Qiagen, Hilden, Germay, 331502) according to the manufacturer’s instructions. Samples derived from six peanut-allergic patients (allergic adults before and one hour after reaction during the oral food challenge) were sequenced using the Illumina Platform.^10^,^11^,^12^

miRNAs were quantified by reverse transcription quantitative real-time PCR (RT-qPCR). In addition, during miRNA isolation from serum, as described above, we added 3.5 μl miRNeasy serum/plasma spike-in control (Qiagen, Hilden, Germany, 219610) at 1.6 x 10^8^ copies/μl. The total RNA was reverse transcribed using miRCURY LNA RNA kit (Qiagen, 339340, Hilden, Germany) that generates universal cDNA templates for all miRNAs present in the sample. The second synthetic spike-in (UniSp6, Qiagen, Hilden, Germany, 339340) was added to each sample and the reaction was performed on a GeneTouch thermal cycler (Bioer, Hangzhou, China). Then, miRNA-specific quantification was performed using the miRCURY LNA SYBR Green kit (Qiagen, Hilden, Germany, 339347) according to the manufacturer’s instructions. The expression of target miRNAs was normalized to the mean of cel-miR-39-3p and UniSp6 synthetic spike-ins added during total RNA extraction and reverse transcription. A two-tailed Student’s t-test for paired data was used for comparing values with an expected normal distribution, utilizing Holm’s p-value correction for multiple comparisons where appropriate. *P*‑values < 0.05 were considered significant.

**Mast Cell Activation Test**

Mast cell activation of PSCMCs was measured as CD63 receptor upregulation (as previously described^13^).

PSCMCs were generated according to Luo et al.^14^ In brief, CD34+ cells were isolated from buffy coats (DRK, Dresden) using the CD34 positive Selection Kit II (STEMCELL Technologies, Vancouver, Canada). CD34+ stem cells were expanded for one week in StemSpan™ SFEM (STEMCELL Technologies, Vancouver, Canada) supplemented with 10% StemSpan CD34+ Expansion Supplement (STEMCELL Technologies, Vancouver, Canada) and 1% Penicillin/Streptomycin and then differentiated into mast cells using StemSpan™ SFEM containing 20 ng/ml IL-3 (BioLegend, San Diego, CA, USA), 50 ng/ml SCF (Miltenyi Biotec., Bergisch-Gladbach, Germany) and 1% Penicillin/Streptomycin. After four weeks, mast cells were purified using the CD117 MicroBead Kit human (Miltenyi Biotec., Bergisch-Gladbach, Germany). PSCMCs were cultured in StemSpan™ SFEM containing 50 ng/ml IL-6 (Peprotech, Hamburg, Germany) and 125 ng/ml SCF and 1% Penicillin/Streptomycin.

To measure mast cell activation, 50000 mast cells were either sensitized with 10% patient serum or 1.18 µg/ml human IgE (myeloma, Merck Millipore, Burlington, MA, USA) and incubated overnight at 37 °C. The next day, cells were washed and stimulated with 0.1 µg/ml and 0.01 µg/ml peanut extract or 1 µg/ml anti-human IgE antibody (rabbit polyclonal, Bethyl laboratories, Montgomery, TX, USA) in SFEM for 1h at 37 °C. To measure receptor upregulation after stimulation, the cells were washed and incubated with Fc-receptor blocking solution (1:50, Human TruStain FcX^™^, BioLegend, San Diego, CA, USA) for 5min. Afterwards the cells were stained with CD63 antibody (clone: H5C6, BioLegend, San Diego, CA, USA) for 30 min, washed three times and stained with DAPI (Roche, Basel, Switzerland). CD63 upregulation was analyzed using a MACSQuant flow analyzer according to the gating strategy illustrated in **Supplementary Figure S7**.

**Supplementary Tables**

**Table S1: Subject characteristics.** Peanut allergy was confirmed in individuals with a history of systemic allergic reactions after peanut consumption through double-blind placebo-controlled food challenges. The non-allergic individuals had no known food allergies and tolerated the ingestion of the cumulative dose of 4.5 g peanut protein. AD – atopic dermatitis, n.d. – not determined, n.a. – not applicable, sIgE - specific IgE to peanut extract or Ara h 2, ^1^Threshold - maximum cumulative threshold dose (g peanut protein) leading to discontinuation of oral food challenge, ^2^Severity score according to modified Sampson Score.

| **ID** | **Age  (years)** | **Sex** | **Atopy** | | | | **Tryptase (µg/l)** | **SPT (mm)** | **IgE  (kU/l)** | | | **Peanut challenge** | |  |
| --- | --- | --- | --- | --- | --- | --- | --- | --- | --- | --- | --- | --- | --- | --- |
|  |  |  | **AD** | | **Asthma** | **Rhinitis** | **Basal** | **Peanut native** | **Total** | **sIgE**  **peanut** | **sIgE Ara h 2** | **Threshold^1^** | **Severity score^2^** |  |
|  | | | | **Allergic** | | | | | | | | | | |
| A1 | 26 | m | no | | no | yes | 3.93 | 7.0 | 38.2 | 0.73 | 1.97 | 0.012 | IV |  |
| A2 | 27 | f | no | | no | no | 4.01 | 11.0 | 238.0 | 74.20 | 68.7 | 0.043 | III |  |
| A3 | 31 | m | yes | | yes | yes | 5.81 | 6.5 | 212.0 | 0.93 | 0.54 | 1.378 | II |  |
| A4 | 28 | f | yes | | no | no | 4.84 | 8.5 | 205.0 | 86.10 | 29.20 | 0.137 | II |  |
| A5 | 34 | m | yes | | yes | yes | 4.53 | 5.0 | 164.0 | 1.27 | 0.58 | 0.043 | III |  |
| A6 | 29 | f | yes | | yes | yes | 3.74 | 9.5 | 66.6 | 9.03 | 8.18 | 0.447 | IV |  |
|  | | | | **Non-Allergic** | | | | | | | | | | |
| NA1 | 35 | f | no | | no | no | 5.88 | 0 | 16.6 | n.d. | n.d. | n.a. | n.a. |  |
| NA2 | 33 | m | no | | no | no | 4.89 | 0 | 144.0 | n.d. | n.d. | n.a. | n.a. |  |
| NA3 | 30 | f | no | | no | no | 4.12 | 0 | 15.2 | n.d. | n.d. | n.a. | n.a. |  |
| NA4 | 30 | f | no | | no | no | 5.51 | 0 | 92.1 | n.d. | n.d. | n.a. | n.a. |  |
| NA5 | 32 | f | no | | yes | no | 6.59 | 0 | 20.0 | n.d. | n.d. | n.a. | n.a. |  |
| NA6 | 32 | f | no | | no | no | 3.89 | 0 | 9.2 | n.d. | n.d. | n.a. | n.a. |  |
| NA7 | 35 | f | no | | no | no | 5.66 | 0 | 469.0 | n.d. | n.d. | n.a. | n.a. |  |

**Table S2: Sampling and sample distribution within this study**

| **Mechanistic subproject** | **Biosample collected** | **Time point** | **Storage step** | **Method** |
| --- | --- | --- | --- | --- |
| **Epigenetics** | Whole blood  (Sodium heparin) | Before the oral food challenge | PBMC, frozen | DNA Isolation and CpG methylation screening |
| **Microbiome** | Stool  (OMNIGgene GUT) | Before the oral food challenge | Frozen | DNA isolation and shotgun sequencing |
| **Adaptive Immunity** | Whole blood (Lithium heparin) | Before the oral food challenge | PBMC, fresh | Allergen-reactive B&T cell phenotyping |
| **Cellular stratification** | Whole blood (Lithium heparin) | Before the oral food challenge | Fresh | Basophil activation test and basophil phenotyping |
| **Allergen-specific IgE** | Whole blood (Serum) | Before the oral food challenge | Serum, frozen | Mast cell activation test |
| **Soluble biomarkers** | Whole blood  (Serum) | Before and 1h after the oral food challenge (in case of reactions 1h after reaction started) | Serum, frozen | ELISA and miRNA sequencing |

**Table S3: Significantly associated differentially methylated regions (DMRs) as well as significantly associated and candidate CpG probes comparing non-allergic and allergic samples** either under (A) non-stimulated or (B) stimulated conditions. Start position, end position and width of the DMRs are indicated. ^a^ CpG probe instead of DMR. ^b^ DMRs/CpGs overlapping coding genes (bold) or GREAT annotation (non-bold), showing the closest genes with transcriptional start site (TSS) either upstream (-) or downstream (+) of respective DMR/CpG (units - bp distance to site). ^c^ P-value is a Šidák-corrected for DMRs and uncorrected for CpG probes; genome-wide significant p-values indicated in bold. ^d^ methylation with respect to allergic cases as used in **Figure 1**, where “+” indicates hypermethylation and “-” indicates hypomethylation in allergic cases. Chr, chromosome; bp, basepairs.

| Chromosome | Start | End | DMR width (bp) or CpG probe^a^ | No. of CpGs | **Gene(s)** or closest TSS^b^ | p-value^c^ | Methlyation direction^d^ |
| --- | --- | --- | --- | --- | --- | --- | --- |
| **(A) Non-stimulated samples: non-allergic versus allergic** | | | | | | | |
| chr19 | 3480363 | 3480672 | 309 | 6 | ***SMIM24*** | **4.27E-07** | + |
| chr10 | 135278901 | 135279147 | 246 | 4 | ***SCART1*** | **2.36E-04** | + |
| chr6 | 31939085 | 31939322 | 237 | 8 | ***STK19;DXO*** | **3.38E-04** | + |
| chr5 | 178986372 | 178986728 | 356 | 9 | ***RUFY1*** | **4.35E-04** | - |
| chr17 | 41278179 | 41278622 | 443 | 9 | ***BRCA1;NBR2*** | **9.20E-04** | + |
| chr3 | 169530621 | 169530875 | 254 | 6 | ***LRRC34*** | **2.08E-03** | - |
| chr10 | 134150451 | 134150700 | 249 | 7 | ***LRRC27*** | **3.70E-03** | - |
| chr13 | 76444798 | 76445015 | 217 | 5 | ***LMO7DN*** | **5.35E-03** | + |
| chr3 | 66848608 | 66848765 | 157 | 4 | *KBTBD8 (-200044), LRIG1 (297331)* | **9.76E-03** | - |
| chr8 | 17433694 | 17433926 | 232 | 6 | ***PDGFRL*** | **3.77E-02** | - |
| chr7 | 135457413 | 135457413 | cg23586565 | 1 | *FAM180A(-23820), MTPN (204651)* | **7.29E-09** | + |
| chr21 | 46349496 | 46349496 | cg02464073 | 1 | ***ITGB2*** | 1.29E-07 | + |
| chr5 | 153588790 | 153588790 | cg04997435 | 1 | ***GALNT10*** | 2.96E-06 | + |
| chr9 | 95862983 | 95862983 | cg01627676 | 1 | ***CARD19*** | 8.30E-06 | + |
| chr4 | 149074093 | 149074093 | cg13000004 | 1 | ***NR3C2*** | 9.57E-06 | + |
| chr2 | 63099116 | 63099116 | cg13692650 | 1 | ***EHBP1*** | 9.92E-06 | + |
| **(B) Stimulated samples: non-allergic versus allergic** | | | | | | | |
| chr19 | 3480363 | 3480672 | 309 | 6 | ***SMIM24*** | **1.23E-07** | + |
| chr5 | 178986291 | 178986906 | 615 | 12 | ***RUFY1*** | **4.64E-07** | - |
| chr10 | 135278901 | 135279147 | 246 | 4 | ***SCART1*** | **1.48E-06** | + |
| chr10 | 134150451 | 134150760 | 309 | 8 | ***LRRC27*** | **1.85E-06** | - |
| chr17 | 41278135 | 41278444 | 309 | 10 | ***BRCA1;NBR2*** | **5.52E-04** | + |
| chr3 | 158390329 | 158390525 | 196 | 5 | ***LXN; GFM1*** | **4.51E-03** | - |
| chr3 | 169530692 | 169530920 | 228 | 5 | ***LRRC34*** | **6.03E-03** | - |
| chr13 | 108866993 | 108867154 | 161 | 4 | ***LIG4*** | **6.06E-03** | - |
| chr13 | 21900392 | 21900591 | 199 | 4 | ZDHHC20 (132931), MRPL57 (149708) | **1.36E-02** | - |
| chr3 | 66848608 | 66848765 | 157 | 4 | *KBTBD8 (-200044), LRIG1 (297331)* | **3.08E-02** | - |
| chr20 | 43936853 | 43936981 | 128 | 4 | ***MATN4; RBPJL*** | **4.26E-02** | - |
| chr7 | 135457413 | 135457413 | cg23586565 | 1 | *FAM180A(-23820), MTPN (204651)* | 3.97E-06 | **+** |
| chr21 | 46349496 | 46349496 | cg02464073 | 1 | ***ITGB2*** | 3.01E-07 | + |
| chr17 | 48915829 | 48915829 | cg26449328 | 1 | ***WFIKKN2*** | 8.75E-07 | - |
| chr11 | 64479015 | 64479015 | cg10940462 | 1 | ***NRXN2*** | 2.07E-06 | + |
| chr4 | 1542171 | 1542171 | cg01409693 | 1 | *NKX1-1 (-142053), FAM53A (143817)* | 5.49E-06 | - |
| chr2 | 175498353 | 175498353 | cg02200333 | 1 | ***WIPF1*** | 5.60E-06 | + |
| chr3 | 106176366 | 106176366 | cg04075990 | 1 | *CBLB(-588480)* | 5.66E-06 | - |
| chr5 | 27532684 | 27532684 | cg24851859 | 1 | *CDH9(-493992)* | 6.66E-06 | - |
| chr12 | 102162134 | 102162134 | cg08298696 | 1 | ***GNPTAB*** | 7.24E-06 | - |
| chr22 | 37414442 | 37414442 | cg17575915 | 1 | ***MPST*** | 7.86E-06 | + |
| chr20 | 51811572 | 51811572 | cg24632232 | 1 | ***TSHZ2*** | 8.47E-06 | - |
| chr14 | 62161200 | 62161200 | cg21072687 | 1 | ***HIF1A-AS1;HIF1A*** | 9.59E-06 | + |

**Table S4: Legend description for Figure 6 the integrative circos plot.**

| **Label** | **Description** | **Feature spaces in the circus plot** |
| --- | --- | --- |
| Allergy status | Clinical characteristic: Allergic (yes) or non-allergic (no) | Grey, Phenotype |
| Peanut-sIgE (kU/l) | Serum level of peanut-specific IgE measured in kU/l. | Grey, Phenotype |
| Basal Tryptase (µg/l) | Serum level of basal tryptase measured in µg/l. | Grey, Phenotype |
| Subjects age | Subject’s age in years at time of enrolment. | Grey, Phenotype |
| Haemophilus | Rarefied microbial abundance on genus level. | Red, Microbiome (genera) |
| unknown Bacteria.1 | Rarefied microbial abundance on genus level. Genus not classified in the database. | Red, Microbiome (genera) |
| unknown Lentisphaerae | Rarefied microbial abundance on genus level. Genus could not be assigned to references in the database. But was classified as Lentisphaerae. | Red, Microbiome (genera) |
| Ruminococcus | Rarefied microbial abundance on genus level. | Red, Microbiome (genera) |
| Chloroflexi | Rarefied microbial abundance on phylum level. | Black, Microbiome (phylum) |
| Bacteroidetes | Rarefied microbial abundance on phylum level. | Black, Microbiome (phylum) |
| unknown phylum Pseudomonadota/ Bacillota | Rarefied microbial abundance on phylum level. Phylum could not be assigned toreferences in the database but classified as Pseudomonodota/Bacilliota. | Black, Microbiome (phylum) |
| Pseudomonadota | Rarefied microbial abundance on phylum level. | Black, Microbiome (phylum) |
| Lentisphaerae | Rarefied microbial abundance on phylum level. | Black, Microbiome (phylum) |
| unknown phylum | Rarefied microbial abundance on phylum level. Phylum could not be assigned to references in the database. | Black, Microbiome (phylum) |
| CgXXX | Methylation levels (β) of significant (*P* < 9 E-8) and candidate (*P* < 1E-5) CpG probes | Green, DNA methylation probes |
| Ara h-specific memory B cells | Percentage of Arah2-specific class-switched memory B cells (CD27+IgD-) in B cells | Blue, Adaptive immunity |
| Peanut-specific Tregs in CD4+ T cells | Percentage of peanut-specific regulatory T cells (CD154-CD137+) in CD4+ T cells | Blue, Adaptive immunity |
| IL5+ in peanut-specific Tconv | Percentage of IL5+ in peanut-specific conventional T cells | Blue, Adaptive immunity |
| IL4+ in peanut-specific Tconv | Percentage of IL4+ in peanut -specific conventional T cells | Blue, Adaptive immunity |
| CCR4+ in peanut-specific Tconv | Percentage of CCR4+ in peanut -specific conventional T cells | Blue, Adaptive immunity |
| Peanut-specific Tconv in CD4+ T cells | Percentage of peanut -specific conventional T cells (CD154+CD137+) in CD4+ T cells | Blue, Adaptive immunity |
| CXCR5+ in peanut-specific Tconv | Percentage of CXCR5+ in peanut-specific conventional T cells | Blue, Adaptive immunity |
| CD33 | Median Fluorescence Intensity of CD33 staining in basophils after stimulation with 1µg/ml peanut extract | Turquoise, Basophil phenotyping |
| CDSENS | Peanut extract concentration in ng/ml required for activation of 50% of the responsive basophils | Turquoise, Basophil phenotyping |
| CDMAX | Percentage of maximum of activated basophils stimulated with peanut extract | Turquoise, Basophil phenotyping |
| pERK | Median Fluorescence Intensity of phospho-ERK 1/2 staining in basophils after stimulation with 1µg/ml peanut extract | Turquoise, Basophil phenotyping |
| pp38 | Median Fluorescence Intensity of phospho-p38 staining in basophils after stimulation with 1µg/ml peanut extract | Turquoise, Basophil phenotyping |
| pSyk | Median Fluorescence Intensity of phospho-Syk staining in basophils after stimulation with 1µg/ml peanut extract | Turquoise, Basophil phenotyping |
| pAKT | Median Fluorescence Intensity of phospho-AKT staining in basophils after stimulation with 1µg/ml peanut extract | Turquoise, Basophil phenotyping |
| CD203c | Median Fluorescence Intensity of CD203c staining in basophils after stimulation with 1µg/ml peanut extract | Turquoise, Basophil phenotyping |
| CD300a | Median Fluorescence Intensity of CD300a staining in basophils after stimulation with 1µg/ml peanut extract | Turquoise, Basophil phenotyping |
| CD63 | Median Fluorescence Intensity of CD63 staining in basophils after stimulation with 1µg/ml peanut extract | Turquoise, Basophil phenotyping |
| Apolipoprotein E (µg/ml) | Level of apolipoprotein E measured in serum | Pink, Soluble biomarkers |
| Arachidonic acid (µg/ml) | Level of arachidonic acid measured in serum | Pink, Soluble biomarkers |
| ECP (µg/ml) | Level of eosinophil cationic protein measured in serum | Pink, Soluble biomarkers |
| MAT using 100ng/ml peanut extract | Percentage of CD63+ PSCMCs sensitized with patient serum after stimulation with 100ng/ml peanut extract | Yellow, Mast cell activation |
| MAT using 100ng/ml peanut extract | Percentage of CD63+ PSCMCs sensitized with patient serum after stimulation with 10ng/ml peanut extract | Yellow, Mast cell activation |

**Table S5:** List of antibodies used for staining of allergen-specific T cells

|  |  |  |
| --- | --- | --- |
| Surface Staining | | |
| CCR4-BV510 | L291H4 | Biolegend |
| CD8-BV650 | RPA-T8 | Biolegend |
| CXCR5-PE-Vio615 | REA103 | Miltenyi |
| CD4-PERCP | RPA-T4 | Biolegend |
|  |  |  |
| Intracellular Staining | | |
| IL-5-BV521 | TRFK5 | Biolegend |
| CD154-BV605 | 24-31 | Biolegend |
| FOXP3-Alexa-Fluor-488 | 259D | Biolegend |
| CD137-PE | REA765 | Miltenyi |
| IL-4-PE-Vio-770 | REA895 | Miltenyi |
| Helios-Alexa-Fluor-647 | 22F6 | Biolegend |
| IFN-g- Alexa-Fluor-700 | 4S.B3 | Biolegend |
| IL-17A-APC-Vio-770 | REA1063 | Miltenyi |

**Table S6:** List of antibodies used for staining of allergen-specific B cells

|  |  |  |
| --- | --- | --- |
| Surface Staining | | |
| IgD-BV510 | IA6-2 | Biolegend |
| CD14-BV570 | M5E2 | Biolegend |
| CD3-BV570 | UCHT1 | Biolegend |
| CD21-BV605 | 1048 | BD bioscience |
| CD23-PE | EBVCS-5 | Biolegend |
| CD27-PE-Dazzle594 | O323 | Biolegend |
| CD24-PercpCy5.5 | ML5 | Biolegend |
| CD19-PE-Cy7 | HIB19 | Biolegend |
| CD38-Alexa-Fluor-700 | HB-7 | Biolegend |
| CD20-APC-Cy7 | 2H7 | Biolegend |
| Ara-H-2 Pacific Blue |  |  |
| Ara-H-2 Alexa-Fluor-488 |  |  |
|  |  |  |
| Intracellular Staining | | |
| Ki67 APC | REA183 | Miltenyi |

**Table S7:** List of antibodies used for staining for basophils phenotying

|  |  |  |
| --- | --- | --- |
| Barcoding | | |
| B2M 89Y | 2M2 | Biolegend |
| B2M 106Cd | 2M2 | Biolegend |
| B2M 110Cd | 2M2 | Biolegend |
| B2M 112Cd | 2M2 | Biolegend |
| B2M 114Cd | 2M2 | Biolegend |
| B2M 116Cd | 2M2 | Biolegend |
|  |  |  |
| Surface staining Before fixation | | |
| CD62L 143Nd | DREG-56 | Biolegend |
| CD63 146Nd | H5C6 | Biolegend |
| CD300a 147Sm | MEM-260 | Invitrogen |
| CD11c 158Gd | REA618 | Miltenyi |
| CD33 159Tb | HIM3-4 | Biolegend |
| CD300c 173Yb | TX45 | Biolegend |
| CD32b 176Yb | 4F5/CD32 | Biolegend |
|  |  |  |
| Surface staining After fixation | | |
| CD1c 141Pr | L161 | Biolegend |
| FCeR1a 142Nd | AER-37 | Biolegend |
| CD15 141Nd | W6D3 | Biolegend |
| CD141 145Nd | REA674 | Miltenyi |
| CD203c 149Sm | FR3-1611 | Miltenyi |
| CD123 151Eu | 6H6 | Biolegend |
| CD14 155Gd | M5E2 | Biolegend |
| CD28 160Gd | CD28.2 | Biolegend |
| CD56 163Dy | HCD56 | Biolegend |
| Siglec8 164Dy | 7C9 | Biolegend |
| CD64 165Ho | 10.1 | Biolegend |
| CD19 166Er | HIB19 | Biolegend |
| CD163 169Tm | REA812 | Miltenyi |
| CD3 170Er | UCHT1 | Biolegend |
| CD24 171Yb | ML5 | Biolegend |
| CD38 172Yb | HIT2 | Biolegend |
| HLADR 174Yb | L243 | Biolegend |
| CD23 175Lu | M-L23.4 | Biolegend |
| CD16 209Bi | REA423 | Miltenyi |
|  |  |  |
| Intracellular staining | | |
| Syk 150Nd | Syk-01 | Biolegend |
| pAKT 152Sm | REA359 | Miltenyi |
| pSyk 153Eu | 1503310 | Biolegend |
| SHIP1 154Sm | P1C1-A5 | Biolegend |
| pp38 156Gd | A16016A | Biolegend |
| pPLG 161Dy | REA341 | Miltenyi |
| pERK 167Er | 4B11B69 | Biolegend |
| SHP1 168Er | W17240D | Biolegend |

|  |  |  |
| --- | --- | --- |
| Surface before fixation | | |
| CD14-Viogreen | Tük4 | Miltenyi |
| CD11c-BV605 | 3.9 | Biolegend |
| CD63-FITC | REA1055 | Miltenyi |
|  |  |  |
| Surface post fixation | | |
| HLADR-Vioblue | REA805 | Miltenyi |
| CD203c-PE | REA826 | Miltenyi |
| CD123-PE-Vio770 | REA918 | Miltenyi |
| CD16-APC-Vio770 | REA423 | Miltenyi |
|  |  |  |
| Intracellular staining | | |
| S6 pS235/pS236-APC | REA454 | Miltenyi |

**Table S8:** List of antibodies used for staining for basophil activation test (BAT)

**Supplementary Figures**


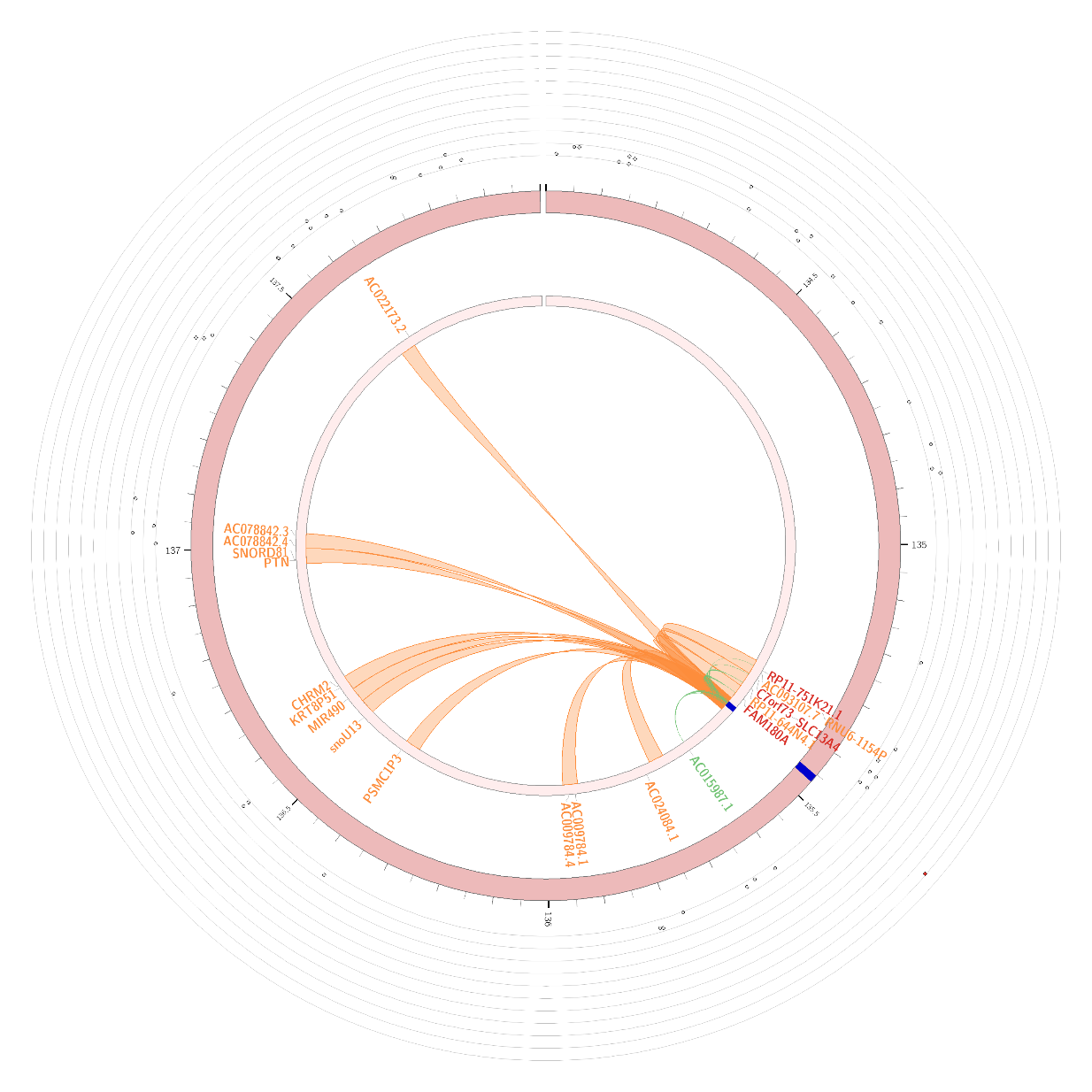
 **Figure S1:** Circos plot indicating eQTLs (green), chromatin interactions (orange), and a combination of both (red) between our locus of interest (blue) containing cg23586565 and other genes in this region. Circos plot is taken from FUMA ^6^.

**
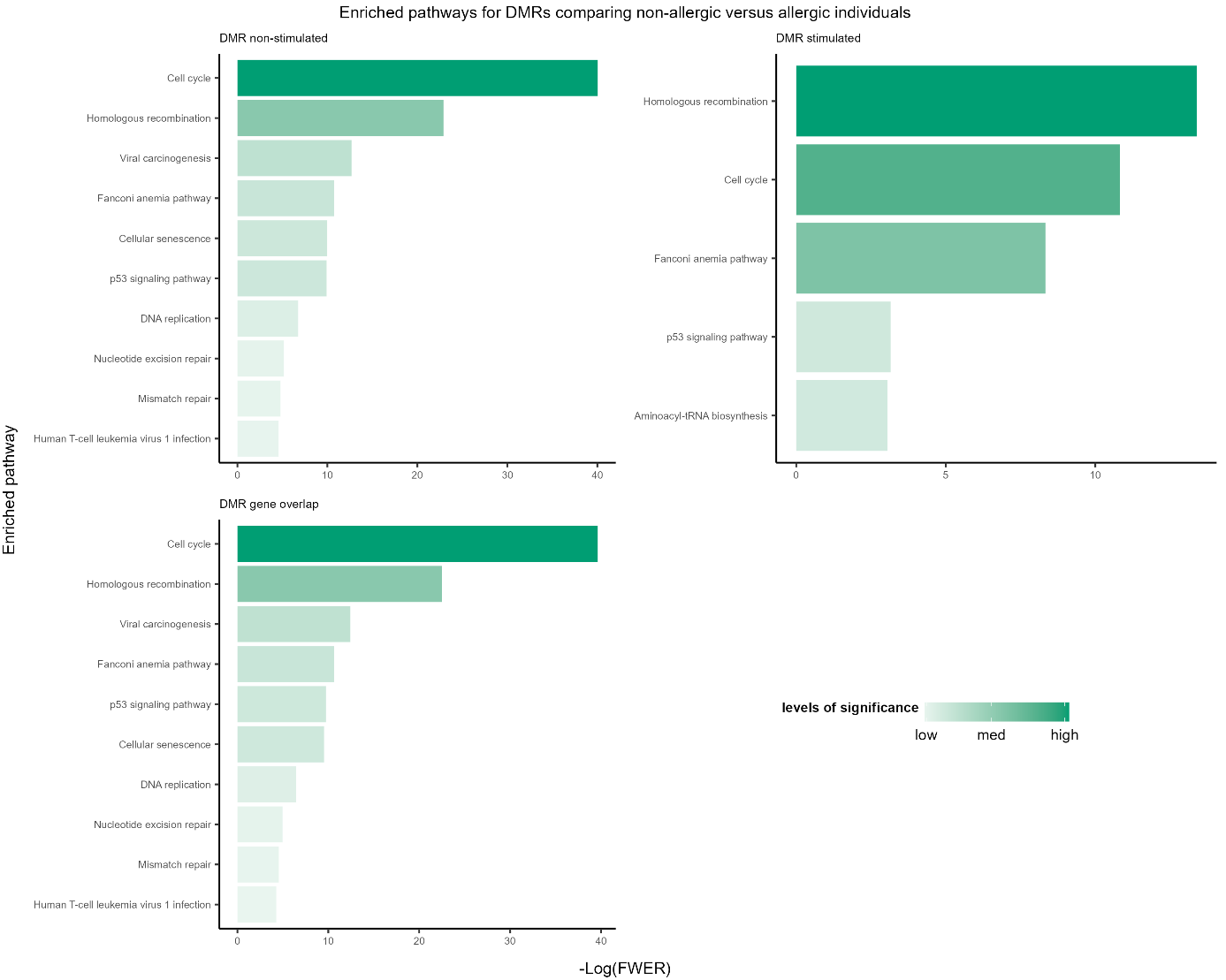
**

**Figure S2:** Enriched pathways for significant DMRs found in the association analysis comparing non-allergic and allergic individuals. Pathway enrichment terms derived from stimulation-specific DMRs as well as overlapping DMRs between analyses are shown.

(B)

(A)


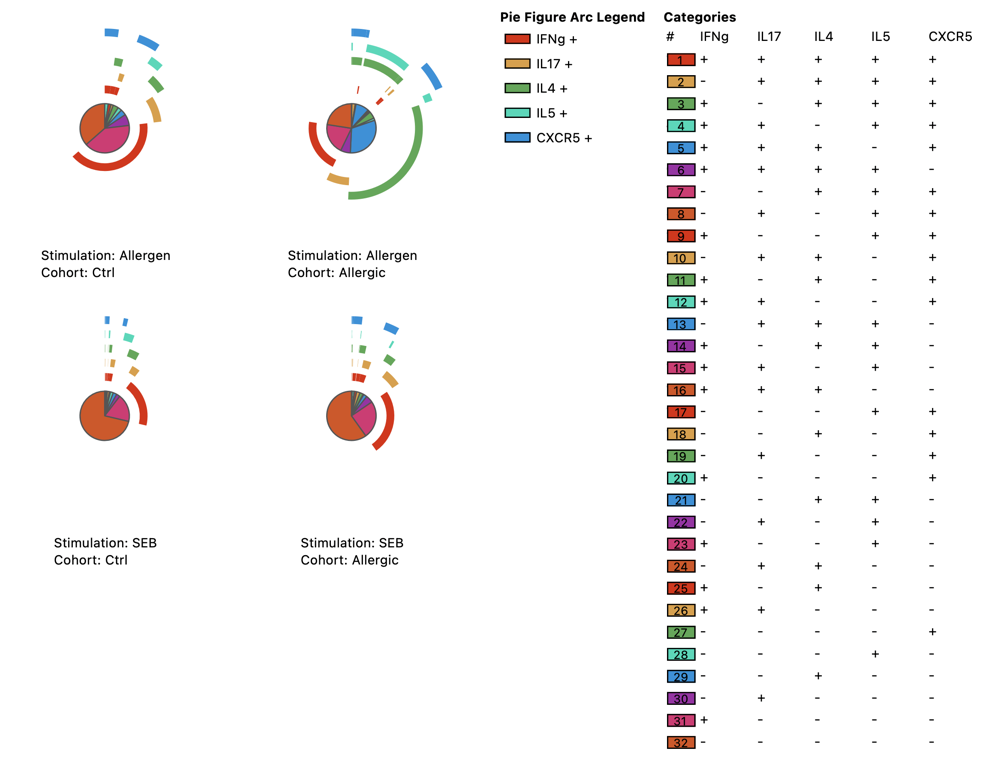


**Figure S3:** Gating strategy for antigen-specific T cells and polyfunctionality analysis: (A) Representative gating example of allergic donor PBMCs stimulated with Peanut extract is shown. Samples were first cleaned for measurement-dependent fluctuations using the Time parameter (HDR-T) before lymphocytes were gated (FSC-A vs SSC-A), from which doublets were excluded via FSC- and SSC- with -H(eight) vs –W(idth). Within alive (Live Dead staining negative) lymphocytes, CD4+ and CD8+ T cells are gated, and within CD4, antigen-reactive CD137+ CD154+ conventional T cells and CD137+ CD154- regulatory T cells are gated. Within conventional antigen-reactive T cells, frequencies of IFNγ, IL17A, IL4, IL5 producing cells as well as CCR4+ and CXCR5+ cells are determined. (B) For the polyfunctionality analysis using SPICE, Boolean combinations of IFNγ, IL17A, IL4, IL5 CCR4 and CXCR5 positive and negative gates are processed to display all frequent and rare combinations (indicated as arcs and pies) of cytokine production and marker expression (Categories).


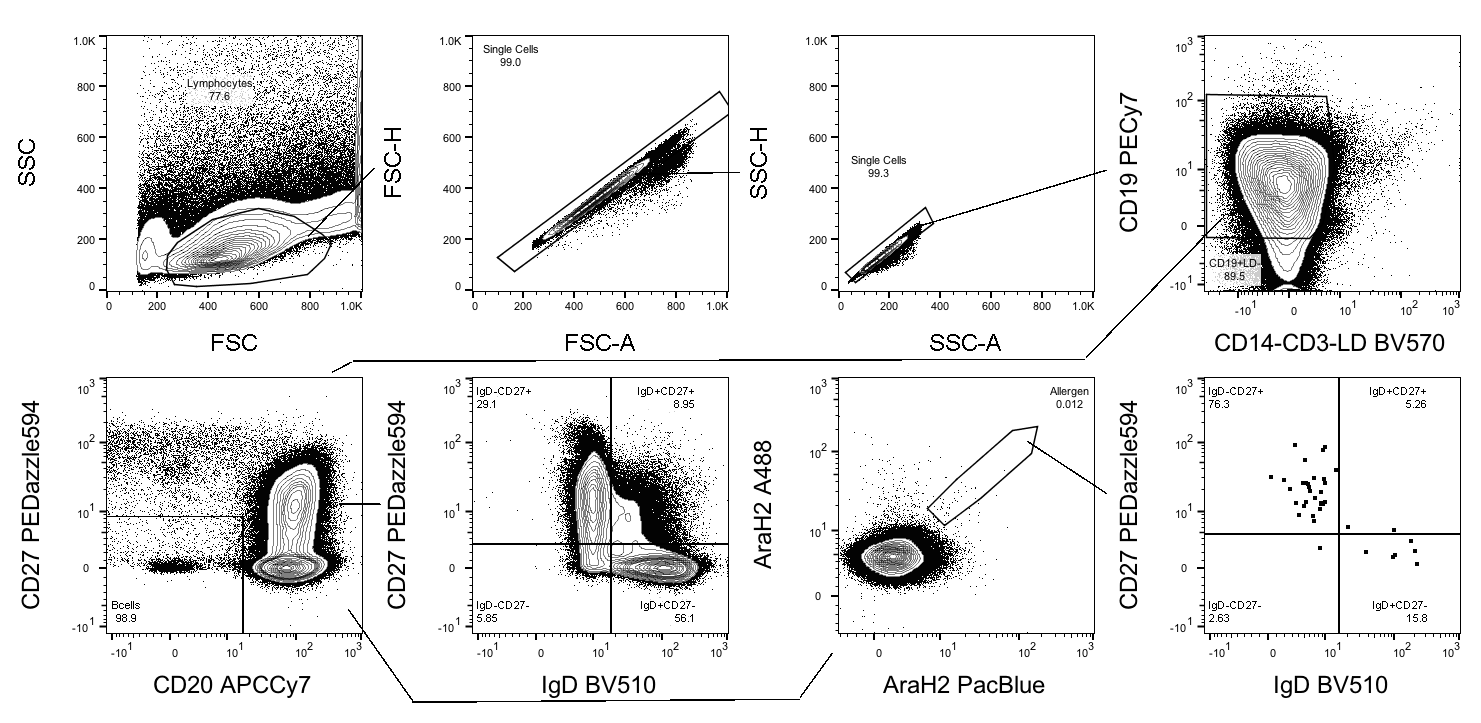


**Figure S4: Gating strategy for antigen-specific B cells** Representative gating example of allergic donor CD19+ enriched stimulated is shown. Lymphocytes were gated (FSC-A vs SSC-A), from which doublets were excluded via FSC- and SSC- with -H(eight) vs –W(idth). Alive (Live Dead staining negative) CD19 + CD3 – CD14 – lymphocytes were gated, and within CD20- CD27- cells were excluded. B cells were further identified using CD27 and IgD, and Ara-H-2 specific B cells were gated, and further characterized with CD27 and IgD.


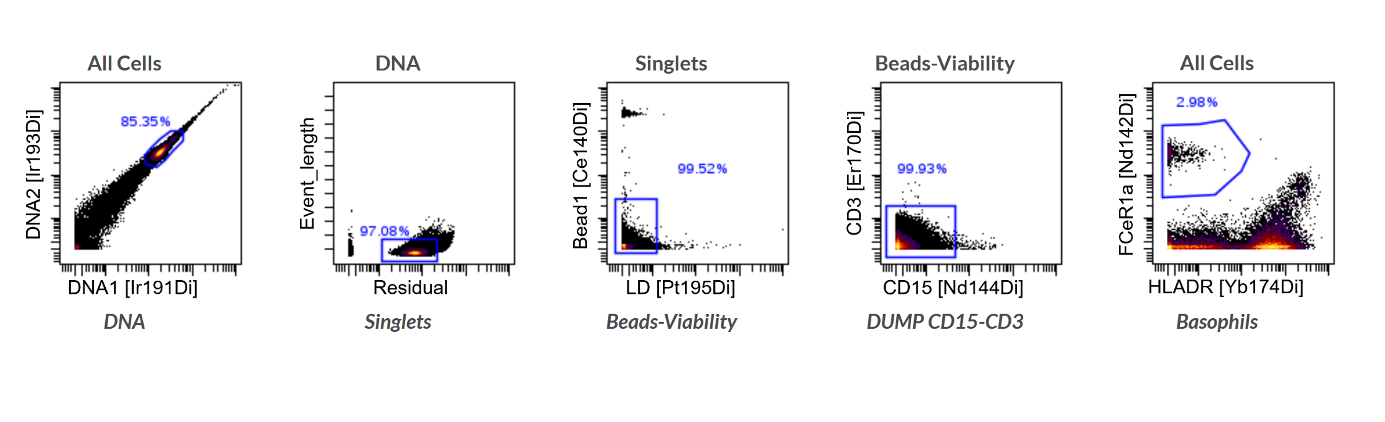


**Figure S5: Basophils phenotyping gating strategy:** Cells were gated on DNA staining, and singlets were identified on Residual and Event length parameters. Live dead were gated using cisplatin intensity and beads used to normalize the mass cytometer during measurements were excluded. CD15+ and CD3+ cells were then excluded, and Basophils were identified as FCER1a + HLADR –


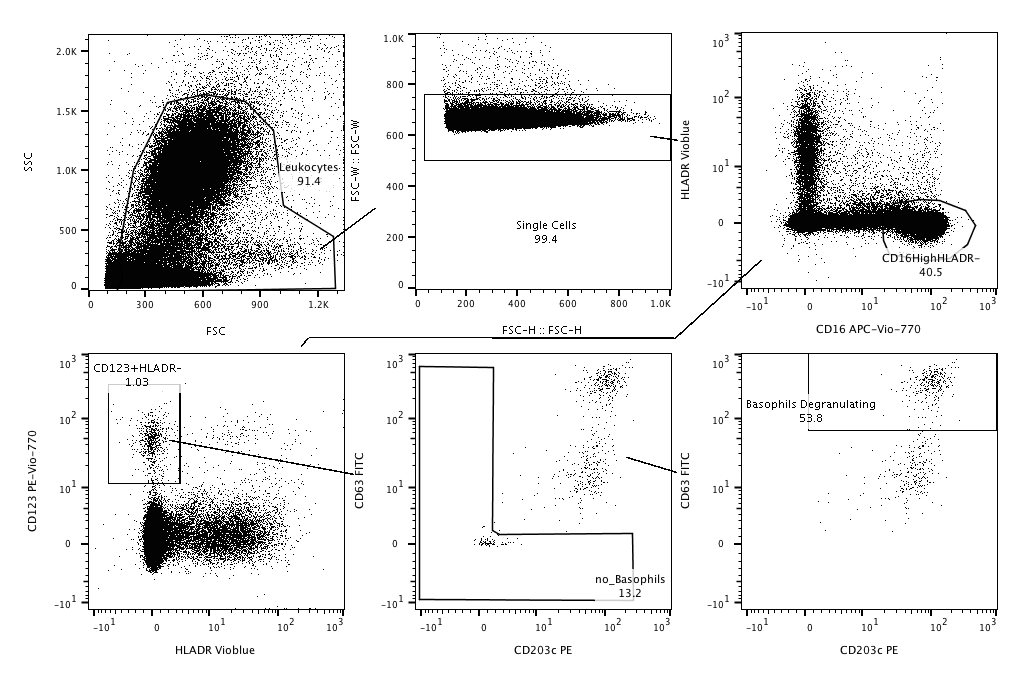


**Figure S6: Basophils activation test gating strategy:** Leukocytes and single cells were gated using FSC and SSC parameters. Then CD16 high HLA-DR- cells were gated out to exclude neutrophils, and basophils were identified as CD123+ HLADR-. CD203c- CD63- cells were excluded to obtain a pure basophils population. Activated and degranulating basophils were identified as CD63 high.


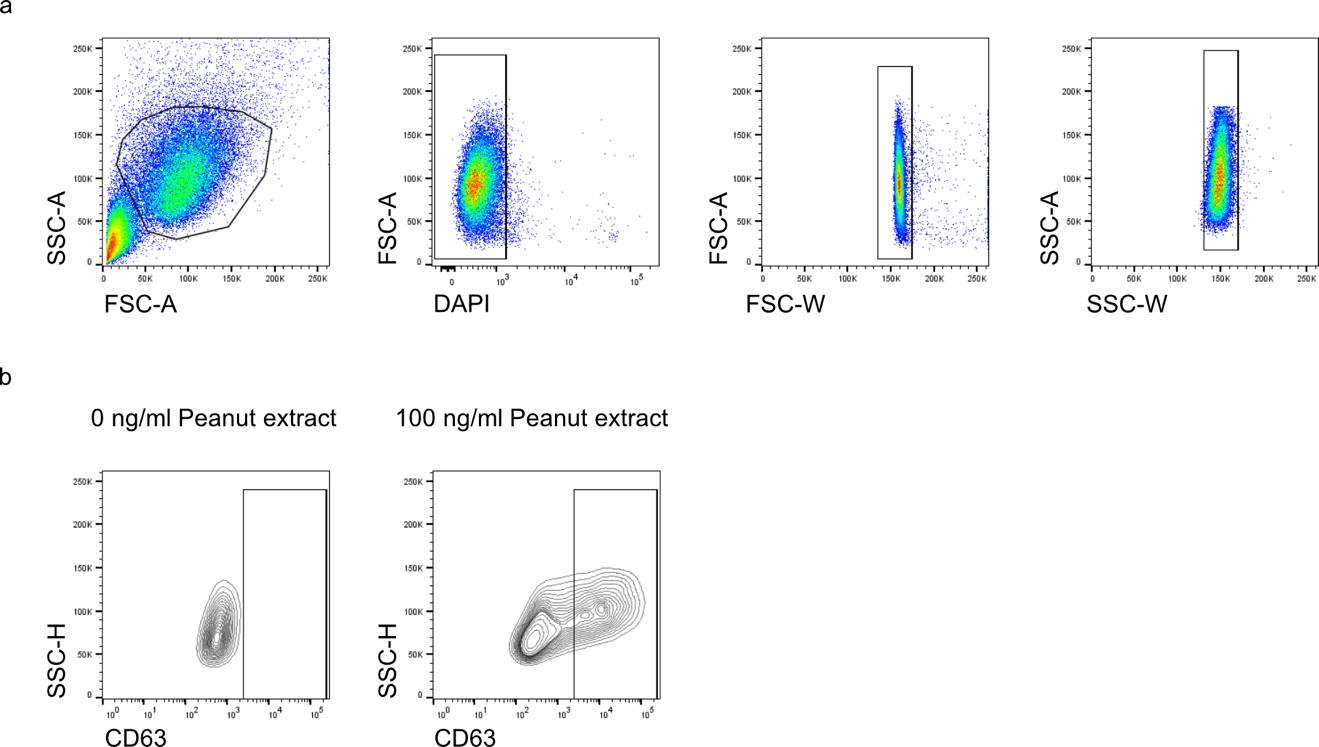


**Figure S7: Mast cell activation test gating strategy:** Representative flow plots. a) PSCMCs were gated using FSC and SSC parameters to isolate from debris and aggregates. After gating on alive cells, single cells were gated using FSC-A versus FSC-W and SSC-A versus SSC-W. b) Activated and degranulating mast cells were identified as CD63+ cells.

**Supplementary Reference list**

1. Min JL, Hemani G, Davey Smith G, Relton C, Suderman M. Meffil: efficient normalization and analysis of very large DNA methylation datasets. *Bioinformatics*. 2018;34(23):3983-9.

2. Mansell G, Gorrie-Stone TJ, Bao Y, et al. Guidance for DNA methylation studies: statistical insights from the Illumina EPIC array. *BMC Genomics*. 2019;20(1):366.

3. Pedersen BS, Schwartz DA, Yang IV, Kechris KJ. Comb-p: software for combining, analyzing, grouping and correcting spatially correlated P-values. *Bioinformatics*. 2012;28(22):2986-8.

4. McLean CY, Bristor D, Hiller M, et al. GREAT improves functional interpretation of cis-regulatory regions. *Nat Biotechnol*. 2010;28(5):495-501.

5. Ogris C, Helleday T, Sonnhammer EL. PathwAX: a web server for network crosstalk based pathway annotation. *Nucleic Acids Res*. 2016;44(W1):W105-9.

6. Watanabe K, Taskesen E, van Bochoven A, Posthuma D. Functional mapping and annotation of genetic associations with FUMA. *Nat Commun*. 2017;8(1):1826.

7. Maifeld A, Bartolomaeus H, Lober U, et al. Fasting alters the gut microbiome reducing blood pressure and body weight in metabolic syndrome patients. *Nat Commun*. 2021;12(1):1970.

8. Coelho LP, Alves R, Monteiro P, Huerta-Cepas J, Freitas AT, Bork P. NG-meta-profiler: fast processing of metagenomes using NGLess, a domain-specific language. *Microbiome*. 2019;7(1):84.

9. Milanese A, Mende DR, Paoli L, et al. Microbial abundance, activity and population genomic profiling with mOTUs2. *Nat Commun*. 2019;10(1):1014.

10. Martin M. Cutadapt Removes Adapter Sequences From High-Throughput Sequencing Reads. *EMBnet Journal*. 2010;17(1):10-2.

11. Griffiths-Jones S, Saini HK, van Dongen S, Enright AJ. miRBase: tools for microRNA genomics. *Nucleic Acids Res*. 2008;36(Database issue):D154-8.

12. Friedlander MR, Mackowiak SD, Li N, Chen W, Rajewsky N. miRDeep2 accurately identifies known and hundreds of novel microRNA genes in seven animal clades. *Nucleic Acids Res*. 2012;40(1):37-52.

13. Bahri R, Bulfone-Paus S. Mast Cell Activation Test (MAT). *Methods Mol Biol*. 2020;2163:227-38.

14. Luo Y, Frischbutter S, Scheffel J, Siebenhaar F. Generation and Culture of Peripheral CD34(+) Stem Cell-Derived Mast Cells (PSCMCs). *Methods Mol Biol*. 2020;2163:63-7.
